# Genetic patterns in Neotropical Magnolias (Magnoliaceae) using *de novo* developed microsatellite markers

**DOI:** 10.1038/s41437-018-0151-5

**Published:** 2018-10-27

**Authors:** Emily Veltjen, Pieter Asselman, Majela Hernández Rodríguez, Alejandro Palmarola Bejerano, Ernesto Testé Lozano, Luis Roberto González Torres, Paul Goetghebeur, Isabel Larridon, Marie-Stéphanie Samain

**Affiliations:** 1 Research Group Spermatophytes, Department of Biology, Ghent University, K.L. Ledeganckstraat 35, Gent, 9000 Belgium; 2 Botanic Garden Meise, Nieuwelaan 38, Meise, 1860 Belgium; 3 Departamento de Biología Vegetal, Facultad de Biología, Universidad de La Habana, C/ 25 e/ I y J, Vedado, La Habana, Cuba; 4 Grupo de Ecología y Conservación, Jardín Botánico Nacional, Universidad de La Habana, Carretera “El Rocio” km 3 ½, Boyeros, La Habana, Cuba; 5 The University of British Columbia, 2329 West Mall, Vancouver, BC V6T 1Z4 Canada; 6 Royal Botanic Gardens, Kew, Surrey, Richmond, TW9 3AE UK; 7 Red de Diversidad Biológica del Occidente Mexicano, Instituto de Ecología, A.C., Avenida Lázaro Cárdenas 253, Pátzcuaro, Michoacán 61600 Mexico

**Keywords:** Genetic variation, Speciation, Genetic markers, Natural variation in plants

## Abstract

Conserving tree populations safeguards forests since they represent key elements of the ecosystem. The genetic characteristics underlying the evolutionary success of the tree growth form: high genetic diversity, extensive gene flow and strong species integrity, contribute to their survival in terms of adaptability. However, different biological and landscape contexts challenge these characteristics. This study employs 63 *de novo* developed microsatellite or SSR (Single Sequence Repeat) markers in different datasets of nine Neotropical *Magnolia* species. The genetic patterns of these protogynous, insect-pollinated tree species occurring in fragmented, highly-disturbed landscapes were investigated. Datasets containing a total of 340 individuals were tested for their genetic structure and degree of inbreeding. Analyses for genetic structure depicted structuring between species, i.e. strong species integrity. Within the species, all but one population pair were considered moderate to highly differentiated, i.e. no indication of extensive gene flow between populations. No overall correlation was observed between genetic and geographic distance of the pairwise species’ populations. In contrast to the pronounced genetic structure, there was no evidence of inbreeding within the populations, suggesting mechanisms favouring cross pollination and/or selection for more genetically diverse, heterozygous offspring. In conclusion, the data illustrate that the Neotropical Magnolias in the context of a fragmented landscape still have ample gene flow within populations, yet little gene flow between populations.

## Introduction

Conservation genetics utilises a representative sample of DNA and organisms to quantify and study genetic diversity to preserve species as dynamic entities capable of coping with environmental change (Frankham et al. 2010). A collection of DNA fragments representing the genome is realised by employing molecular markers: fragments of DNA associated with a certain location within the genome, providing information about the allelic variation at the given locus (Schlötterer 2004). Microsatellite or SSR (Simple Sequence Repeat) markers are often the preferred type of molecular marker in conservation genetics because they are codominant, highly polymorphic, ubiquitous, reproducible and neutral; and they have a high mutation rate, as well as an easy sample preparation (Selkoe and Toonen 2006). Although it is labour and cost intensive to develop and test SSR primer pairs, these can often be employed across species, with success decreasing proportionally to relatedness (Kalia et al. 2011). A representative sampling of organisms can be interpreted at different levels: individuals for populations, populations for species, and species for ecosystems. The latter strategy makes use of the umbrella species concept (Roberge and Angelstam 2004).

An exemplar group of umbrella species are trees: they maintain the structure and function of forest ecosystems, and create resource niches and patches for other organisms (Pautasso 2009). Trees also provide various ecosystem services and resources for human use (Neale and Kremer 2011) and their genetics and evolution have paradoxical features (Petit and Hampe 2006). Trees were found to maintain high levels of genetic diversity (Hamrick et al. 1992), but experience low nucleotide substitution rates and low speciation rates when compared to annual plant lineages (e.g. Bousquet et al. 1992; Petit and Hampe 2006; Whittle and Johnston 2003). They combine high local differentiation for adaptive traits (Aitken et al. 2008) with extensive gene flow (Austerlitz et al. 2000; Kremer and Le Corre 2012). Furthermore, they maintain species integrity, while expressing abundant interspecific gene flow (Ellstrand et al. 1996). The abovementioned features provide an expected capacity for tree survival, as they create resilience against threats such as climate change or habitat fragmentation (Aitken et al. 2008; Hamrick 2004). However, the interplay of the biological and landscape context challenges these generalised characteristics and creates the need for context-oriented tree conservation genetic studies and subsequent management guidelines (Aparicio et al. 2012; Dick et al. 2008).

To investigate the general patterns of tree genetics in an empirical setting, and to contribute to the conservation of the species and forests under study, we focus on New World representatives of the tree genus *Magnolia* (Magnoliaceae) occurring at tropical latitudes, hereafter named Neotropical Magnolias. *Magnolia* trees provide an interesting case-study with bisexual, protogynous flowers, specialised beetle pollination with tepal movement, variable flowering phenology and seed dispersal by animals (Thien 1974). The Red List of Magnoliaceae (Rivers et al. 2016) states that 76% of the Neotropical Magnolias are threatened, with an additional 16% listed as data deficient. Neotropical *Magnolia* populations have not been studied from a molecular point of view (Cires et al. 2013) and their species are delineated based on morphological and distributional argumentation (e.g. Howard 1948; Palmarola et al. 2016; Vázquez-García et al. 2013b). Many of the *Magnolia* species and populations occur in fragmented, highly-disturbed, relict primary forest landscapes, such as the cloud forests of the Caribbean islands and the cloud and rain forests of Mexico (Rivers et al. 2016).

This study aims to (1) provide *de novo* developed SSR markers for Neotropical *Magnolia* species; (2) employ the SSR markers for genetic species delimitation between Caribbean *Magnolia* species; (3) search for patterns of extensive gene flow between Caribbean *Magnolia* (sub)species and populations; and (4) test for signs of inbreeding within the Neotropical *Magnolia* populations.

## Material and methods

### Sampling and DNA extraction

**Fig. 1:**
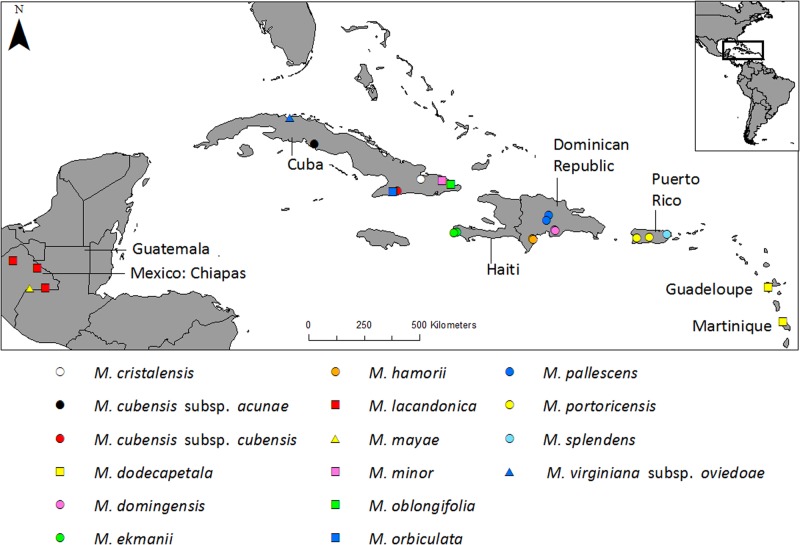
Location map of 16 *Magnolia* taxa (i.e. 15 *Magnolia* species, of which one species consists of two subspecies) from the Caribbean and Mexico, collected in the wild. Circles represent the species of the section *Talauma* subsection *Splendentes*. Squares represent species of the *Talauma* subsection *Talauma*. Triangles represent species of the section *Magnolia*. Classification is according to Figlar and Nooteboom (2004)

Sample information of the 17 different taxa (i.e. 16 species, of which one species consists of two subspecies) and 17 populations included in this study are given in Table 1. A map, showing the location information of the wild collected accessions of Neotropical *Magnolia* from the Caribbean and Mexico, is given in Fig. 1. The wild collected samples comprise 346 samples, of which 340 represent the 17 populations. The additional six wild collected samples represent single collections of different species. One further sample is from an *ex situ* collection of *M. dealbata*.

**Table 1:** Sample information of 17 *Magnolia* taxa (i.e. 16 species, of which one species consists of two subspecies) and 17 populations included in the SSR testing and/or genotyping

Taxa	Tax.	Population	Pop.	Class.	Country	RL	Herbarium reference
*M. cristalensis*	CRI	–	–	TAS	Cuba	EN	Falcón et al. HFC-88423 (HAJB)
*M. cubensis* subsp. acunae*	ACU	Topes de Collantes	TOP	TAS	Cuba	CR	Palmarola & González-Torres HFC-89432 (HAJB)
*M. cubensis* subsp. *cubensis*	CUB	Pico Turquino	PIC	TAS	Cuba	VU	Palmarola & González-Torres HFC-89418 (HAJB)
M. dealbata*	DEA	**–**	**–**	MAC	Mexico	NT	Veltjen 2018-001 (Arboretum Wespelaar)
*M. dodecapetala*	DOD	Martinique	MART	TAT	Lesser Antilles	VU	Veltjen et al. 2016-010 (GENT, K, MTK)
		Guadeloupe	GUA				Veltjen et al. 2016-015 (GENT, GUAD)
*M. domingensis*	DOM	Loma Barbacoa	BAR	TAS	Hispaniola	CR	Veltjen et al. 2015-011 (GENT, JBSD)
		Loma Rodríguez	ROD				Veltjen et al. 2015-012 (GENT, HAJB, JBSD)
*M. ekmanii*	EKM	Morne Grand Bois	GRA	TAS	Haiti	CR	Veltjen et al. 2015-001 (EHH, IEB, GENT)
		Morne Mansinte	MAN				Veltjen et al. 2015-003 (EHH, IEB, GENT, JBSD, K)
*M. hamorii*	HAM	Cortico	COR	TAS	Dominican Republic	E	Veltjen et al. 2015-009 (GENT, HAJB, JBSD, K)
		Cachote	CAC				Veltjen et al. 2015-010 (GENT, JBSD)
M. lacandonica*	LAC	Lacanjá	LAC	TAT	Mexico	CR	Samain et al. 2013-039 (IEB, MEXU)
		Yajalón	YAJ				Samain & Martínez 2017-016 (IEB, MEXU)
M. mayae*	MAY	–	–	MAG	Mexico	CR	Samain 2013-048 (IEB, MEXU)
*M. minor*	MIN	–	–	TAT	Cuba	EN	Palmarola et al. HFC-84609 (HAJB)
*M. oblongifolia*	OBL	–	–	TAT	Cuba	CR	Falcón et al. HFC-89377 (HAJB)
*M. orbiculata*	ORB	–	–	TAT	Cuba	VU	Palmarola & González-Torres HFC-89393 (HAJB)
*M. pallescens*	PAL	Loma de la Sal	SAL	TAS	Dominican Republic	E	Veltjen et al. 2015-004 (GENT, JBSD)
		Montellano	MON				Veltjen et al. 2015-007 (GENT, JBSD)
*M. portoricensis*	POR	Toro Negro	TOR	TAS	Puerto Rico	E	Veltjen & Rodríguez-Guzmán 2015-015 (GENT, K, UPRRP)
		Maricao	MARI				Veltjen 2015-016 (GENT, UPRRP)
*M. splendens*	SPL	El Yunque	YUN	TAS	Puerto Rico	E	Veltjen et al. 2015-013 (GENT, UPRRP)
*M. virginiana*	VIR	–	–	MAG	US	LC	Conrad s.n. (GENT)

The four taxa used for microsatellite marker development are denoted with an asterisk. **Taxa** according to García-Morales et al. (2017); González Torres et al. (2016); Howard (1948); Vázquez-García et al. (2013a) and Vázquez-García et al. (2013b). **Tax**.: three letter code to represent the (sub)species. **Pop**.: three or four letter code to represent the population. When there is no population code this means that only one DNA sample was present, used for amplification testing only. **Class**.: classification according to Figlar and Nooteboom (2004); **MAC**: section *Macrophylla*; **MAG**: section *Magnolia*; **TAS**: section *Talauma* subsection *Splendentes*; **TAT**: section *Talauma* subsection *Talauma*. **RL**: Red List status according to González Torres et al. (2016) and Rivers et al. (2016); **CR**: Critically Endangered; **E**: Endangered. **VU**: Vulnerable. All three (i.e. E, CR and VU) Red List statuses are considered to be threatened. Herbarium acronyms are according to the Index Herbariorum (Thiers, [continuously updated]). Samples were collected in 2013 (Mexico, Cuba), 2014 (Cuba), April-May 2015 (Hispaniola, Puerto Rico), June 2016 (Lesser Antilles), August-October 2016 (Puerto Rico) and February 2017 (Mexico)

For the 17 populations included in the full genetic analyses, Average Pairwise Distance between individuals (APD), Maximum distance between consecutive individuals (Max), Spatial extent of the populations (SpE) and number of sampled individuals per populations (N_S_) are given in Table 2. Pairwise distances were calculated using the fossil package (Vavrek 2011) in R v.3.4.3 (R Core Team 2016).

**Table 2:** Population statistics of Caribbean and Mexican Magnolias

Tax.	Pop.	N_S_	SpE	Max	APD	M		P		N_G_		A		H_o_		H_e_		F_is_	
						T	S	T	S	T	S	T	S	T	S	T	S	T	S
ACU	TOP	20	3.78	1.8	1.44	31	10	69.565	90	19.871	20	5.452	5.9	0.594	0.610	0.591	0.647	0.021	0.083
CUB	PIC	20	5.32	3.9	1.85	30	10	70.455	100	19.967	20	5.833	6.6	0.597	0.625	0.613	0.674	0.052	0.098
DOD	MART	20	17.92	10.2	8.62	21	–	65.517	–	19.857	–	6.714	–	0.451	–	0.528	–	0.170*	–
DOD	GUA	20	26.08	10.4	12.39	21	–	68.966	–	19.905	–	7.238	–	0.515	–	0.573	–	0.127*	–
DOM	BAR	20	0.16	0.05	0.06	19	10	62.500	100	19.947	20	4.263	5.4	0.625	0.750	0.573	0.673	–0.065	–0.089
DOM	ROD	20	0.28	0.09	0.10	19	10	62.500	100	20.000	20	3.368	3.8	0.503	0.600	0.482	0.577	–0.018	–0.014
EKM	GRA	20	1.02	0.28	0.47	28	10	57.447	100	20.000	20	4.536	4.3	0.482	0.520	0.464	0.496	–0.013	–0.024
EKM	MAN	20	1.52	0.88	0.40	28	10	59.574	80	19.929	19.9	3.786	3.4	0.475	0.465	0.458	0.449	–0.012	–0.01
HAM	COR	20	0.98	0.79	0.15	22	10	60.000	90	20.000	20	6.682	6.2	0.723	0.650	0.712	0.668	0.011	0.053
HAM	CAC	20	1.70	0.60	0.71	22	10	60.000	90	20.000	20	6.591	6.5	0.707	0.635	0.704	0.661	0.021	0.064
LAC	LAC	20	–	–	–	20	–	64.706	–	20.000	–	4.500	–	0.638	–	0.603	–	–0.032	–
LAC	YAJ	20	0,23	0.81	0.10	20	–	67.647	–	20.000	–	4.750	–	0.688	–	0.592	–	–0.135	–
PAL	SAL	20	0.62	0.19	0.20	18	10	59.375	100	20.000	20	4.611	5.5	0.514	0.625	0.511	0.638	0.021	0.046
PAL	MON	20	0.16	0.05	0.05	18	10	59.375	100	20.000	20	4.278	5.2	0.464	0.580	0.483	0.594	0.066	0.049
POR	TOR	20	10.45	6.1	3.43	28	10	70.000	100	20.000	20	6.286	6.4	0.525	0.510	0.607	0.625	0.160*	0.209*
POR	MARI	20	1.95	1.4	0.90	28	10	67.500	90	19.964	20	5.357	6.0	0.566	0.645	0.564	0.622	0.022	–0.011
SPL	YUN	20	8.08	3.7	3.31	23	10	69.444	100	19.957	20	5.391	6.2	0.580	0.630	0.602	0.662	0.063	0.073

**Tax**.: abbreviations of (sub)species according to Table 1. **Pop**.: population abbreviations according to Table 1. **N**_**S**_: number of sampled individuals. **SpE**: Spatial Extent (in km): the greatest pairwise distance in the population. **Max**: Maximum distance (in km) between two consecutive individuals of a population (i.e. with no other (recorded) individual(s) in between). **APD**: Average Pairwise Distance between individuals (in km). **M**: number of microsatellite markers employed. **T**: taxon-datasets, which include all the markers out of the 63 published microsatellite markers that were polymorphic and unambiguous to score for the species at hand (Supplementary Table S2: A), omitting the markers with high probability of containing null alleles (Supplementary Table S4). **S**: The *Splendentes*-normalized dataset (dataset 3) which contain ten microsatellite markers that could be genotyped for all the 8 taxa of the section *Talauma* subsection *Splendentes* (Figlar and Nooteboom 2004) present in this study (See Supplementary Table S2: all the microsatellite markers indicated with an asterisk). **P**: percentage of polymorphic loci (%). **N**_**G**_: average number of genotyped individuals. **A**: average number of alleles. **H**_**o**_: average observed heterozygosity. **H**_**e**_: average expected heterozygosity. **F**_**IS**_: population inbreeding coefficient, significant deviations from Hardy-Weinberg proportions are indicated with * (*p* = 0.05)

All 347 leaf samples were dried in silica gel and their DNA was isolated using a modified cetyltrimethylammonium bromide (CTAB) (Doyle and Doyle 1987) extraction protocol, with MagAttract Suspension G solution (Qiagen, Germantown, USA) (Xin and Chen 2012) mediated cleaning (Larridon et al. 2015). DNA quantity and quality control was executed using a Qubit® 2.0 Fluorometer (Thermo Fisher Scientific, Massachusetts, USA) and Nanodrop 2000 Spectrophotometer (Thermo Fisher Scientific), respectively.

### SSR markers: development and testing

Primer pairs were developed to amplify sequences containing SSR repeats based on four Neotropical *Magnolia* species: *Magnolia lacandonica* (MA39), *M. mayae* (MA40), *M. dealbata* (MA41), and *M. cubensis* subsp. *acunae* (MA42). The development of the enriched microsatellite library was outsourced to Allgenetics® (A Coruña, Spain) where enrichment was performed using the Nextera XT DNA kit probes (Illumina, California, USA) with the following motifs: AGG, ACG, AAG, AAC, ACAC and ATCT. The library was sequenced on an Illumina MiSeq® platform.

From the 4 × 500 predetermined SSR primer pairs provided by Allgenetics®, 176 were selected for further testing: 49 developed from MA39-reads, 20 developed from MA40-reads, 20 developed from MA41-reads and 87 developed from MA42-reads. Selection of the 176 SSR markers was carried out randomly, respecting the characteristics specified in Guichoux et al. (2011). The forward primers were linked with a universal tail to accomplish multiplex pooling in a three-primer PCR (Vartia et al. 2014). The following universal tags were used: T3: 5′ AATTAACCCTCACTAAAGGG 3′, M13(-20): 5′ GTAAAACGACGGCCAGT 3’, Hill: 5’ TGACCGGCAGCAAAATTG 3′ (Tozaki et al. 2001) and Neomycin reverse: 5′ AGGTGAGATGACAGGAGATC 3’. The reverse primers had a PIG-tail (Brownstein et al. 1996).

All 176 markers were screened for amplification success on the 17 taxa, each represented by one randomly selected sample. PCRs were performed on a total volume of 13 µL under the following conditions: 2 min at 95 °C; 35 cycles of 95 °C for 30 s, 52 °C for 30 s, 72 °C for 90 s; 72 °C for 6 min. The Master Mix contained 0.2 µM forward primer, 0.2 µM reverse primer, 5 ng/ml DNA (suspended in 1 × TE buffer), 1 × TrueStart Taq Buffer (Thermo Fisher Scientific), 1.5 µM MgCl_2_ (Thermo Fisher Scientific), 0.125 µM dNTP, 5U of TrueStart Hot Start DNA polymerase (Thermo Fisher Scientific), and 0.4 mg/ml BSA (bovine serum albumin) per reaction. PCR products were run on a 1% agarose gel, stained with ethidium bromide and visualised under UV-light. Every (sub)species × primer combination was scored. Amplification scores of the 63 published SSR markers are given in the Supplementary Table S1. The (sub)species × primer combinations which were scored to have a single band were submitted to polymorphism testing.

Polymorphism tests were executed on eight individuals per *Magnolia* species, comprising four individuals per predefined population. The individuals for the test-multiplexes were selected to be spatially spread throughout the populations and have 260/230 and 260/280 OD (Optical Density) ratios approximating 2. The (sub)species × primer combinations were scored: 63 were considered polymorphic and unambiguous SSR markers in at least one of the ten tested taxa (Supplementary Table S2). These 63 SSR markers were used for species-specific multiplex design and final genotyping. Their primer information can be found in Supplementary Table S3.

Genotyping of individuals was executed by a multiplex pooling with a three-primer PCR (Vartia et al. 2014). The fluorescent labels FAM, NED, PET and VIC were linked to the tails T3, Hill, Neo and M13, respectively. The multiplex pools were designed using Multiplex Manager (Holleley and Geerts 2009). Multiplex PCRs were performed on a total volume of 5 µL, under the following conditions: 15 min at 95 °C; 35 cycles of 94 °C for 30 s, 57 °C for 90 s, 72 °C for 90 s; 72 °C for 10 min. Each multiplex reaction contained 2 × QIA Multiplex PCR Master Mix (Qiagen), 5 ng/µL DNA, 0.025 µM for each forward primer, 0.1 µM for each reverse primer and 0.1 µM for each specified dye, carrying the same universal tail as the selected forward primer of the chosen primer pairs. Fragment analyses were executed by Macrogen Inc. (Seoul, South Korea) on an ABI 3730XL fragment analyser (Thermo Fisher Scientific) with a GeneScan^TM^ 500 LIZ^TM^ ladder (Thermo Fisher Scientific). The results were analysed in Geneious v.8.1.9 (http://www.geneious.com, Kearse et al. 2012) using the microsatellite plugin. When the test on the subset of individuals appeared promising (i.e. one set of clear peaks, good amplification and more than one allele), 20 individuals per population were genotyped for that marker. The ten taxa were genotyped for 21–36 polymorphic markers, delivering ten separate taxon-datasets (Supplementary Table S2: one taxon-dataset = one column with the markers coded “A”).

Error rates (Selkoe and Toonen 2006) for the markers (Supplementary Table S3) across all ten taxon-datasets were calculated, but were not actively and consistently tested for: duplicate genotyping was produced as a side-product during testing for polymorphism, optimizing multiplexes, re-genotyping a complete multiplex for (a) low/unclear peak(s), or as positive control between PCR batches.

The ten taxon-datasets were submitted to MICRO-CHECKER v.2.2.3 (Van Oosterhout et al. 2004) and ML-NullFreq (Kalinowski and Taper 2006) to test for null alleles. MICRO-CHECKER was run with 1000, and ML-NullFreq was run with 100 000 repetitions. Based on the results, markers with a high probability of representing null alleles were discarded from all downstream analyses.

To ensure that all amplified genetic regions were independent samples of the genome, allelic associations (Lewontin and Kojima 1960) (synonym: Linkage Disequilibrium = LD) per population were analysed in each of the ten taxon-datasets using the software program GENEPOP v.4.3 (Rousset 2008) with the dememorization number set to 10 000, batches set to 1000 and 50 000 iterations per batch. Evaluation of allelic associations was executed by examining both the uncorrected (Waples 2015) and (sequential Bonferroni) corrected p-values (Holm 1979) with nominal p-values of 0.05 per species and per population.

### Genetic structure

To assess the utility of the SSR markers for genetic species delimitation between closely located Caribbean *Magnolia* species and to search for patterns of extensive gene flow between Caribbean *Magnolia* (sub)species, five different supraspecific (i.e. above species level) datasets were instated. Dataset 1 comprises 340 individuals representing 17 populations, genotyped for all their polymorphic and monomorphic loci (see Supplementary Table S2: all marker × taxon combinations coded A, B and C). Hence, for this dataset it was assumed that the loci that tested to be monomorphic for four or eight individuals were monomorphic for all 20 individuals. Dataset 2 comprises 340 individuals representing 17 populations, genotyped for all the polymorphic and monomorphic loci, but not the assumed monomorphic loci (See Supplementary Table S2: all marker × taxon combinations coded A and B). Dataset 3, or the *Splendentes*-normalized-dataset, comprises ten loci (see Supplementary Table S2: SSR markers labelled with an asterisk) that were genotyped for 260 individuals representing 13 populations and eight taxa of section *Talauma* subsection *Splendentes* (Table 1: Class. = TAS). Added to datasets 1, 2 and 3, two smaller supraspecific datasets were instated, representing the apparently closely related species i.e. the two species of Puerto Rico: the PR-dataset; and the three species of the Dominican Republic: the DR-dataset. To search for patterns of extensive gene flow between Caribbean *Magnolia* population pairs within the defined species, the 17 populations were studied on the infraspecific (i.e. below species) level using nine species-datasets (i.e. the taxon-datasets of the two *M. cubensis* subspecies were joined) and 17 population-datasets.

A first batch of analyses was conducted in STRUCTURE v.2.3.4 (Pritchard et al. 2000) on datasets 1, 2 and 3, the PR- and DR-datasets, the nine species-datasets and the 17 population-datasets. STRUCTURE analyses were run with a burn-in of 100 000, 100 000 MCMC steps after the burn-in and the admixture model as ancestry model. Datasets 1, 2 and 3 were run with the allele frequency model set to independent allele frequencies. They were expected to consist of 13 (dataset 3) or 17 (dataset 1 and 2) populations and were run with K set from 1 to 25. The PR- and DR-datasets were run both with the independent allele frequency model and the correlated allele frequency model and their results were compared. They were expected to have between 2 and 6 populations and K was set from 1 to 15. The nine species-datasets and 17 population-datasets were run with the allele frequency model set to correlated allele frequencies. They were run with K set from 1 to 10. For all datasets, each value of K was run 10 times. The results were visualized with Structure Harvester Web v.0.6.94 (Earl and vonHoldt 2012). The best K-value was selected using the ΔK statistic (Evanno et al. 2005) and the results for mean maximum likelihood (Mean LnK). The latter was taken into consideration because the ΔK statistic appointed K-values with unstable replicate results for datasets 1, 2 and 3 and because the ΔK statistic cannot detect single clusters: an outcome expected at the infraspecific level (i.e. population-datasets and possibly the species-datasets). Barplots were visualised using DISTRUCT v.1.1 (Rosenberg 2004).

DAPC analyses (Discriminant Analysis of Principal Components) on datasets 1, 2 and 3 were executed in R using the package adegenet (Jombart 2008). In the find.clusters function we retained 300 PCs for dataset 1 and 2, and 140 PCs for dataset 3. The number of PCs to retain for the PCA eigenvalues was determined using cross-validation. All discriminant functions (DA eigenvalues) were kept.

Pairwise F_ST_ values (Weir and Cockerham 1984) and their confidence intervals were calculated in R using the package diveRsity (Keenan et al. 2013). To visualize the genetic distances for dataset 1, 2 and 3, an unrooted network applying the Neighbour-joining (NJ) method based on Nei’s genetic distance: D_A_ (Nei et al. 1983), was constructed using Populations v.1.2.32 (http://bioinformatics.org/populations/) using 1000 bootstrap replicates as a confidence measure.

Mantel tests on the supraspecific level were performed in GenAlEx v.6.5 (Peakall and Smouse 2006; Peakall and Smouse 2012) on the pairwise log-transformed geographic distance and pairwise F_ST_ values using 9999 permutations. Coordinates of one individual were taken as a representative of its population. Species geographic distance was averaged over the populations of the species.

### Inbreeding and population statistics

To test for inbreeding within the Caribbean *Magnolia* populations, the inbreeding coefficient (F_IS_) for each locus and population was calculated in FSTAT. Tests to detect significant deviations from Hardy-Weinberg proportions (HWP) were calculated in GENEPOP, performing 2-tailed exact tests for each locus in each population. Complete enumeration was performed whenever possible (Louis and Dempster 1987), otherwise MCMC chains were run with 200 batches and 50 000 iterations (Guo and Thompson 1992). Deviations of both the uncorrected and sequential Bonferroni corrected p-values were used to evaluate if populations were truly deviating from HWP (Waples 2015). To frame and discuss the results, different statistical parameters were calculated for each locus and population within the ten taxon-datasets using GenAlEx, i.e. the percentage of polymorphic loci (P), the number of genotyped individuals (N), mean number of alleles (A), expected heterozygosity (H_e_), and observed heterozygosity (H_o_).

## Results

### SSR markers

Overall, 82–92% of the primer pairs amplified, of which 53–67% were scored to be a single amplification product (Supplementary Table S1). The polymorphism tests of the markers giving a single amplification product classified 16–37% of the primer pairs unambiguous and polymorphic (Supplementary Table S2). The reported SSR primers all have heterozygote states in at least one individual and a perfect motif (Weber 1990). For 56 SSR markers, the duplicate runs rendered the same genotypes (Supplementary Table S3: error rate: 0%). For one SSR marker no genotypes were duplicated. The error rates of the other six SSR markers ranged from 1–3.85%.

Results of detection and frequency of null alleles per marker × population combination are given in Supplementary Table S4. Twelve marker × species combinations were considered to have a high probability of showing null alleles: *M. cubensis* (MA42_028), *M. domingensis* (MA39_199), *M. ekmanii* (MA39_023, MA42_087), *M. hamorii* (MA40_223, MA42_413), *M. lacandonica* (MA39_182), *M. pallescens* (MA39_023, MA42_472), *M. portoricensis* (MA42_481) and *M. splendens* (MA39_023, MA42_481).

Associated alleles per marker × species combination are given in Supplementary Table S4. *Magnolia domingensis* and *M. lacandonica* showed a number of SSR markers with associated alleles that were higher than expected for the number of pairwise tests executed. The other eight taxa fell within their confidence intervals of false positives, whereby one significantly associated pair of SSR markers was detected in *M. pallescens* (MA40_045 × MA42_472).

### Genetic structure: supraspecific level

**Fig. 2:**
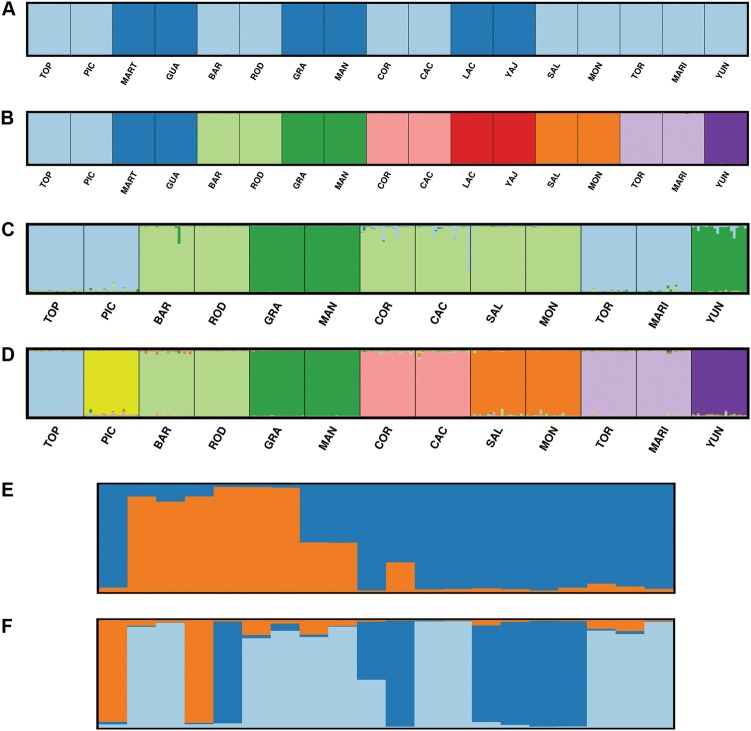
STRUCTURE barplots of Magnolias from the Caribbean and Mexico. The replicate with the highest likelihood score is given. **a** STRUCTURE barplot of dataset 1 and dataset 2, K = 2. **b** STRUCTURE barplot of dataset 1: K = 9. **c** STRUCTURE barplot of dataset 3, K = 3. **d** STRUCTURE barplot of dataset 3, K = 8. **e** STRUCTURE barplot of the Guadeloupe population of *Magnolia dodecapetala*. **f** STRUCTURE barplot of the Toro Negro population of *Magnolia portoricensis*. Dataset 1 comprises 340 individuals representing 17 populations, genotyped for all 63 microsatellite markers where possible, including the assumed monomorphic data (See Supplementary Table S2: categories A, B and C). Dataset 2 comprises 340 individuals representing 17 populations, genotyped for all 63 microsatellite markers where possible, excluding the assumed monomorphic data (See Supplementary Table S2: categories A and B). Dataset 3 comprises 260 individuals representing 13 populations of the 8 taxa of the section *Talauma* subsection *Splendentes* (See Table 1: Class. = TAS), genotyped for 10 microsatellite markers (See Supplementary Table S2: marker names indicated with an asterisk)

**Fig. 3:**
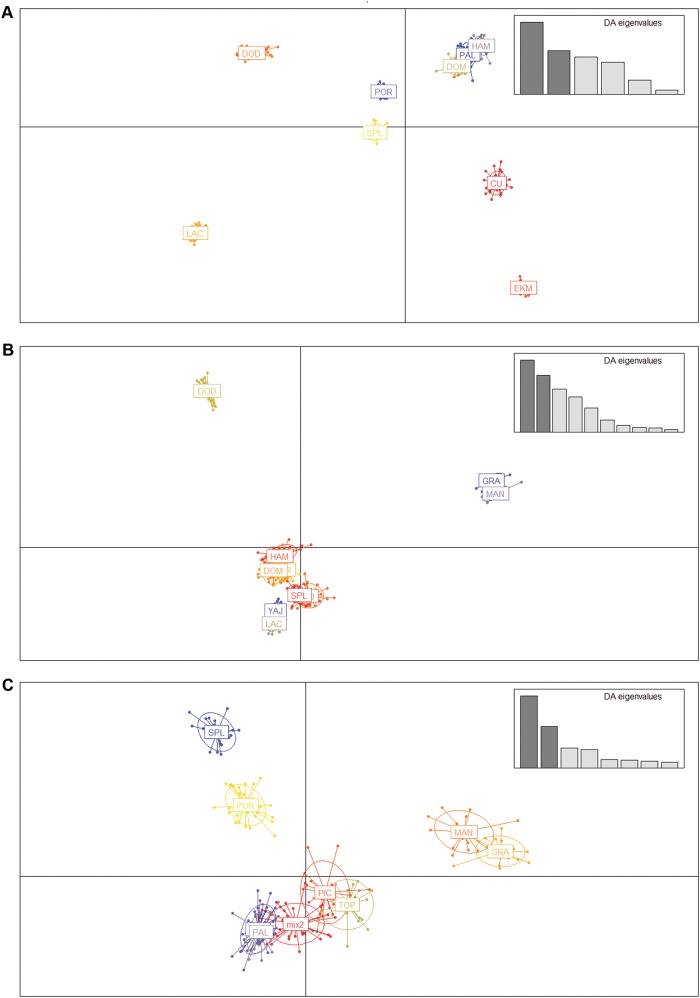
DAPC plots of Magnolias from the Caribbean and Mexico. DAPC: Discriminant Analysis of Principal Components. Populations and (sub)species are abbreviated cf. Table 1 and **CU:** *Magnolia cubensis*. **a** DAPC plot of dataset 1 which comprises 340 individuals representing 17 populations, genotyped for all 63 microsatellite markers where possible, including the assumed monomorphic data (See Supplementary Table S2: categories A, B and C). Nine clusters are visualised following the nine species: CU, DOD, DOM, EKM, HAM, LAC, PAL, POR, SPL **b** DAPC plot of dataset 2 which comprises 340 individuals representing 17 populations, genotyped for all 63 microsatellite markers where possible, excluding the assumed monomorphic data (See Supplementary Table S2: categories A and B). Eleven clusters are visualised: CU (behind SPL), DOD, DOM, HAM, GRA, LAC (population), MAN, PAL (behind POR), POR (behind DOM), SPL, YAJ. **C** DAPC plot of dataset 3 which comprises 260 individuals representing 13 populations of the 8 taxa of the section *Talauma* subsection *Splendentes* (See Table 1: Class. = TAS), genotyped for 10 microsatellite markers (See Supplementary Table S2: marker names indicated with an asterisk). **mix1**: all 40 individuals of DOM and 3 individuals of SAL. **mix2**: all 40 individuals of PAL and 1 individual of PIC. Nine clusters are visualised: GRA, MAN, mix1 (behind PAL), mix2, PAL, PIC, POR, SPL, TOP

**Fig. 4:**
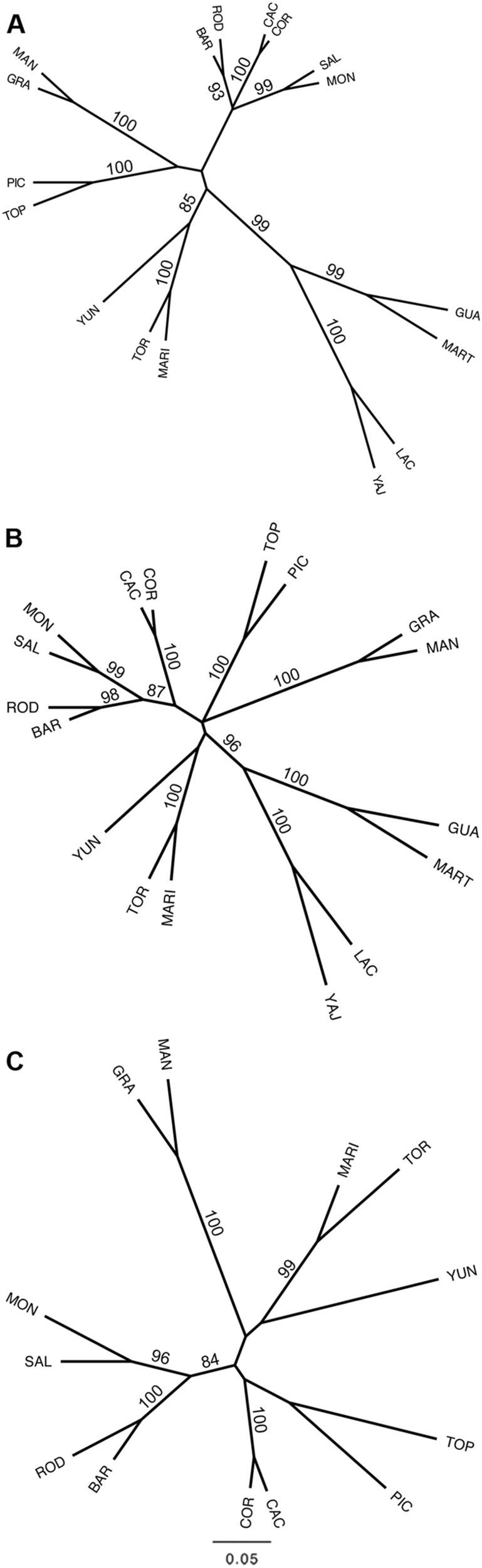
NJ trees of the Magnolias from the Caribbean and Mexico. Unrooted networks are constructed by the Neighbour-joining (NJ) method based on Nei’s genetic distance: D_A_ (Nei et al. 1983). Bootstrap values above 70 are depicted. **a** NJ-tree of dataset 1 which comprises 340 individuals representing 17 populations, genotyped for all 63 microsatellite markers where possible, including the assumed monomorphic data (See Supplementary Table S2: categories A, B and C). **b** NJ-tree of dataset 2 which comprises 340 individuals representing 17 populations, genotyped for all 63 microsatellite markers where possible, excluding the assumed monomorphic data (See Supplementary Table S2: categories A and B). **c** NJ-tree of dataset 3 which comprises 260 individuals representing 13 populations of the 8 taxa of the section *Talauma* subsection *Splendentes* (See Table 1: Class. = TAS), genotyped for 10 microsatellite markers (See Supplementary Table S2: marker names indicated with an asterisk).

Supraspecific ΔK and Mean LnK plots are depicted in Supplementary Figure S5A–E and their interpretation is summarized in Table 3. Barplots of the STRUCTURE analyses on the three full supraspecific datasets are depicted in Fig. 2a–d. The DR-dataset and PR-dataset structured according to the species given both criteria and correlation frequency models. In the DAPC analysis, the “true” K in the replicate runs of the find.clusters algorithm was not univocal, and ranged between 9–13 for dataset 1, 9–15 for dataset 2 and 8–11 for dataset 3. For each dataset, a representative DAPC analysis is visualised in Fig. 3. Supraspecific pairwise F_ST_ values range from 0.216 to 0.618 for dataset 1, 0.166 to 0.472 for dataset 2 and 0.130 to 0.308 for dataset 3 (See Table 4). Their confidence intervals are visualized in Supplementary Figure S6. The unrooted NJ trees based on D_A_ are depicted in Fig. 4. The Mantel tests for all three datasets including all population-pairs were significant (*p* = 0.000–0.003). Mantel tests on the supraspecific pairwise distances were significant for dataset 1 (*p* = 0.000), but not for dataset 2 (*p* = 0.080) and dataset 3 (*p* = 0.256). See Supplementary Figure S7 for visualisation of the relationship between geographic and genetic distance and Table 4 for the Pairwise Geographic Distance (PGD) between the population pairs.

**Table 3:** Number of STRUCTURE clusters of Magnolias from the Caribbean and Mexico

	D1	D2	D3	DR(i)	DR(c)	PR(i)	PR(c)			
ΔK	2	2	3	3	3	2	2			
Mean LnK	9	10	8	7	4	3	3			
S5	A	B	C	D1	D2	E1	E2			

	CU	DOD	DOM	EKM	HAM	LAC	PAL	POR	SPL	
ΔK	2	2	2	2	2	2	2	2	7	
Mean LnK	2	2	3	2	1	2	2	5	1	
S5	F	G	H	I	J	K	L	M	N	

	TOP	PIC	GUA	MART	BAR	ROD	GRA	MAN	CAC	COR
ΔK	2	2	2	2	3	5	2	6	5	2
Mean LnK	1	1	2	1	1	1	1	1	1	1
S5	O1	O2	P1	P2	Q1	Q2	R1	R2	S1	S2

	LAC	YAJ	SAL	MON	MARI	TOR	YUN			
ΔK	7	7	5	8	3	3	7			
Mean LnK	1	1	1	1	1	3	1			
S5	T1	T2	U1	U2	V1	V2	N			

**D1** = dataset 1 which comprises 340 individuals representing 17 populations, genotyped for all 63 microsatellite markers where possible, including the assumed monomorphic data (See Supplementary Table S2: categories A, B and C). **D2** = dataset 2 which comprises 340 individuals representing 17 populations, genotyped for all 63 microsatellite markers where possible, excluding the assumed monomorphic data (See Supplementary Table S2: categories A and B). **D3** = dataset 3 which comprises 260 individuals representing 13 populations of the 8 taxa of the section *Talauma* subsection *Splendentes* (See Table 1: Class. = TAS), genotyped for 10 microsatellite markers (See Supplementary Table S2: marker names indicated with an asterisk). **DR:** DR-dataset comprising the 120 individuals comprising 6 populations and 3 species of the Dominican Republic for all the markers of which data was generated (See Supplementary Table S2: categories A, B and C in the columns DOM, HAM and PAL). **PR**: PR-dataset comprising comprising 60 individuals representing three populations and two species of Puerto Rico for all the markers of which data was generated (See Supplementary Table S2: categories A, B and C in the columns POR and SPL). The DR- and PR-dataset were run with the independent allele model **(i)** and the correlated allele model **(c)**. Abbreviations of species and populations are according to Table 1; **CU**: *Magnolia cubensis*. **ΔK** according to Evanno et al. (2005). **Mean LnK** = Mean maximum likelihood. **S5**: the corresponding plots in Supplementary Figure S5

**Table 4:** Pairwise F_ST_ values and pairwise geographic distance (**PGD** in km) of Magnolias from the Caribbean and Mexico

Sp.		CU	DOD	DOM	EKM	HAM	LAC	PAL	POR	SPL
CU	D1	**0.154**								
	D2	**0.154**								
	D3	**0.160**								
	PGD	**408.404**								
DOD	D1	0.513	**0.181**							
	D2	0.360	**0.181**							
	D3	–	–							
	PGD	1897.652	**168.881**							
DOM	D1	0.428	0.499	**0.138**						
	D2	0.262	0.264	**0.138**						
	D3	0.196	–	**0.093**						
	PGD	890.127	1009.428	**4.540**						
EKM	D1	0.455	0.618	0.486	**0.223**					
	D2	0.387	0.472	0.380	**0.223**					
	D3	0.272	–	0.296	**0.226**					
	PGD	513.501	1418.235	424.854	**10.079**					
HAM	D1	0.389	0.520	0.216	0.497	**0.044**				
	D2	0.187	0.339	0.166	0.325	**0.044**				
	D3	0.130	–	0.132	0.275	**0.035**				
	PGD	817.711	1088.315	100.864	333.286	**3.785**				
LAC	D1	0.539	0.471	0.573	0.611	0.570	**0.185**			
	D2	0.316	0.373	0.318	0.423	0.307	**0.185**			
	D3	–	–	–	–	–	–			
	PGD	1481.214	3245.707	2274.335	1849.511	2181.049	**109.658**			
PAL	D1	0.466	0.557	0.318	0.574	0.279	0.607	**0.163**		
	D2	0.300	0.346	0.230	0.416	0.216	0.283	**0.163**		
	D3	0.152	–	0.164	0.301	0.150	–	**0.115**		
	PGD	843.4194	1057.382	66.576	399.205	114.939	2244.901	**27.064**		
POR	D1	0.409	0.489	0.422	0.535	0.404	0.541	0.534	**0.101**	
	D2	0.246	0.352	0.236	0.396	0.240	0.316	0.314	**0.101**	
	D3	0.152	–	0.226	0.308	0.218	–	0.210	**0.105**	
	PGD	1259.906	647.440	379.509	803.612	471.798	2652.663	418.427	**52.916**	
SPL	D1	0.437	0.559	0.487	0.564	0.461	0.580	0.549	0.338	–
	D2	0.264	0.373	0.237	0.402	0.208	0.266	0.282	0.233	–
	D3	0.227	–	0.226	0.290	0.223	–	0.257	0.239	–
	PGD	1353.569	567.164	479.761	904.498	573.613	2753.896	515.043	102.892	–

**F**_**ST**_ = θ cf. Weir and Cockerham 1984. Species (**Sp**.) are abbreviated cf. Table 1 and **CU** = *Magnolia cubensis*. **D1** = dataset 1 which comprises 340 individuals representing 17 populations, genotyped for all 63 microsatellite markers where possible, including the assumed monomorphic data (See Supplementary Table S2: categories A, B and C). **D2** = dataset 2 which comprises 340 individuals representing 17 populations, genotyped for all 63 microsatellite markers where possible, excluding the assumed monomorphic data (See Supplementary Table S2: categories A and B). **D3** = dataset 3 which comprises 260 individuals representing 13 populations of the 8 taxa of the section *Talauma* subsection *Splendentes* (See Table 1: Class. = TAS), genotyped for 10 microsatellite markers (See Supplementary Table S2: marker names indicated with an asterisk). On the diagonal (in **bold**): the pairwise infraspecific F_ST_ values and the pairwise distances between the pairs of populations per species

### Genetic structure: infraspecific level

Infraspecific ΔK and Mean LnK plots are depicted in Supplementary Figure S5F–V2 and their interpretation is summarized in Table 3. Barplots of the two infraspecific STRUCTURE analyses exceeding the predefined clusters: GUA and TOR are given in Figs. 2e, f, respectively. Infraspecific pairwise F_ST_ values can be found in Table 4 and range from 0.044 to 0.222 for the species-datasets and 0.035 to 0.226 when standardized cf. dataset 3. Confidence intervals of the infraspecific pairwise F_ST_ values are depicted in Supplementary Figure S6. Mantel tests at the infraspecific level were not significant (dataset 1 and dataset 2: *p* = 0.084, dataset 3: *p* = 0.080): see Supplementary Figure S7.

### Inbreeding: infraspecific level

Detailed results on the population statistics calculated on the ten taxon-datasets are listed per marker, population and subset in Supplementary Table S4. Population statistics of the most representative subset are listed in Table 2. Three populations: GUA, MART and TOR showed significant departure from HWP. GUA and MART presented significant deviation from HWP for 5/21 and 4/21 loci (1.45 [0, 3] expected to test false positive when *p* = 0.05). TOR showed significant deviation from HWP for 7/29 loci (1.45 [0, 3] expected to test false positive when *p* = 0.05).

## Discussion

### SSR markers

The data on marker development show an attrition of usable SSR primer pairs during post-sequencing marker development, which is a general issue in SSR development (Hodel et al. 2016). Patterns in success of the polymorphism tests should be treated with caution because (1) multiplexing SSR markers can lead to marker interaction; (2) testing with more individuals or populations can show more markers to be polymorphic; (3) massive parallel testing was executed, for which some SSR marker-species combinations were not replicated; (4) random isolated PCR artefacts have been observed. Because all 63 reported SSR markers had a heterozygous state in at least one individual and contain di- or tri-repeats, they are labelled nuclear SSR loci (Wheeler et al. 2014).

### Sampling design

The sampling design renders a dataset which is standardised yet limited in the number of individuals per population and populations per species (Hoban et al. 2013; Ward and Jasieniuk 2009). It is possible that the limited number of samples invoked false positives or false negatives due to random sampling error (Waples 2015), hence, we recommend including SSR markers that reported to have null alleles when genotyping more individuals and populations in further analyses, except for the markers that have very strong evidence i.e. MA42_028 for *M. cubensis*, MA39_182 for *M. lacandonica* and MA42_481 for *M. portoricensis*.

### Genetic structure: supraspecific level

In general, results of all supraspecific analyses (Tables 3–4, Figs. 2, 3, 4, Supplementary Figure S5A–E2, S6, S7) are influenced by the datasets used. Firstly, due to the resolution: inclusion of more differentiated species/populations conceals the signal of the lower genetic structural levels (e.g. Fig. 2a vs. Fig. 2b). Secondly, due to inclusion or exclusion of the assumed monomorphic SSR loci or fixed alleles (e.g. Fig. 3a vs. Fig. 3b). On the one hand, fixed alleles determined a higher differentiation among species. This is apparent in the NJ-tree when comparing branch lengths and bootstrap values in Figs. 4a, b and in the DAPC plots when comparing Fig. 3a with Fig. 3b. On the other hand, the monomorphic loci strengthen genetically similar species groups, illustrated by the three species of the Dominican Republic to be clustered together in Fig. 4a, while when omitting the assumed monomorphic data (Fig. 4b), *M. hamorii* is differentiated from the other two Dominican Magnolias.

Currently, a molecular phylogenetic analysis including a representative sampling of section *Talauma* and its four subsections (Figlar and Nooteboom 2004; Pérez et al. 2016) is not available. On the basis of the SSR results, it can be stated that the species delineations of the studied seven species of subsection *Splendentes* are genetically confirmed. Clustering methods placed individuals and populations in their respective species genetic cluster (Figs. 2b, d, 3a, b and 4). However, the likelihood of clustering according to the species was not significant enough for the ΔK method to recognize the K corresponding to the number of species (Figs. 2a, c) and species-clusters often overlap in the two-dimensional visualization of the DAPC analysis (Fig. 3) or even consistently cluster with another species (Fig. 3c: mix1, mix2).

Although the SSR data is able to deliver evidence for species boundaries, there can be little conclusions drawn on their evolutionary relationships (Fig. 3, Table 4, Fig. 4). The data illustrates that the set of three Dominican Magnolias and the set of two Puerto Rican Magnolias are the least genetically differentiated (Table 4, Figs. 3a, 4a), which is also visible as a gap in pairwise F_ST_ values (Supplementary Figure S6) and the significant results of the Mantel tests (Supplementary Figure S7). The pairwise F_ST_ values (Table 4, Supplementary Figure S6A, S6B) suggest (*M. domingensis* *+* *M. hamorii*) + *M. pallescens*; however, in Figs. 3, 4b, c (M*. domingensis* *+* *M. pallescens*) + *M. hamorii* is put forward. Although native to the same island as the three Dominican Magnolias, *M. ekmanii* is conspicuously differentiated from them, as well as from all other species. There is a hint that EKM is most closely related to the Cuban Magnolias: their pairwise F_ST_ calculated on dataset 1 is significantly lower compared to the other EKM pairwise comparisons (Table 4, Supplementary Figure S6A), the DAPC analyses (Fig. 3) place them more closely together according to the two most explanatory axes in the ordination space, and the NJ-tree of dataset 1 and 2 display shared ancestry, albeit unsupported (Figs. 4a, b). For EKM and species relationships across the different Caribbean islands, the SSR loci have accumulated too many (homologous) mutations for supported relationships to be deducted (Calonje et al. 2009). Therefore, studying more conservative DNA fragments by phylogenetic studies (e.g. on chloroplast DNA or single copy nuclear genes) would be valuable.

### Genetic structure: infraspecific level

GUA, MART and TOR are suspected to suffer from the Wahlund effect given the larger spatial distances (Table 2: SpE, Max, APD), significantly high number of null alleles (Supplementary Table S4), significant F_IS_ value (Table 2), high number of alleles (Table 2: A) and their population STRUCTURE (Figs. 2e, f). The absence of genetic HWP-based structure in the MART population could be due to unequal mixture fractions (Waples 2015) combined with a small sample size. For more in-depth study of these populations, it is recommended to invoke more substructure in future sampling design and analyses.

The range of pairwise infraspecific F_ST_ values (Table 4) is large (0.035–0.226) and the genetic differentiation can be labelled: little (HAM), moderate (DOM, PAL: dataset 2, POR), great (CUB, DOD, EKM, LAC, PAL: dataset 1*)* (Hartl and Clark 1997) or significant (CUB, DOD, EKM, LAC, PAL: dataset 1) (Frankham et al. 2010). The large range of pairwise, infraspecific F_ST_ values reminds us of the conflict between the continuity of lineage separation and the discrete entity of a species (de Queiroz 1998). Theoretically, infraspecific genetic differentiation was expected to be counteracted by extensive gene flow between populations: either by long-distance pollen dispersal (Petit and Hampe 2006) or seed dispersal by natural disturbances (Lugo et al. 1981).

The Wahlund effect and moderate to great genetic differentiation indicate that the population dynamics of the studied Neotropical Magnolias occur at a fine spatial scale; in this sampling design suggested to be limited in the spatial extent of 4 km (Table 4: PGD of HAM) to 6 km (Table 2: SpE of TOR). The Mantel tests on the infraspecific level (Supplementary Figure S7) and comparisons with *Magnolia* SSR literature (Kikuchi and Isagi 2002; Setsuko et al. 2007; Zhao et al. 2012) show no correlations or trends between pairwise geographic and infraspecific genetic distance. For this result, the biological context (i.e. different animal vectors), different evolutionary histories (i.e. recent long-distance dispersal), and different landscape context (i.e. less fragmented landscapes vs. highly disturbed landscapes) cannot be decoupled from one another. However, given the conservative flower and fruit morphology within the Magnoliaceae family and the extensive deforestation history of the studied populations, the landscape context is expected to be the main driver.

Unexpectedly, the two subspecies of *M. cubensis* express low genetic differentiation combined with a high geographic distance, while we find high structuring overall for the other Magnolias. Here, the hypothesis of relatively recent long-distance dispersal is put forward as the most likely explanation to be tested in further research. Similarly, MAR and GUA, the “populations” of *M. dodecapetala*, were expected to have a higher degree of genetic differentiation compared to the other infraspecific genetic differentiation regardless of the Wahlund effect, given that the populations are separated by ocean and that a “population” on Dominica lies in between that of Guadeloupe and Martinique.

### Population statistics: infraspecific level

The high amount of allelic association found in three populations (ROD, LAC, YAJ) is most likely due to a major reduction in population size: a recent bottleneck. This is concluded given that (a) there is genome-wide allelic association for all three populations, in contradicting strengths when compared across populations pairs per species; and (b) the visited locations had a high degree of disturbance. The samples studied of the ROD and LAC populations indicate that they have not been able to recombine their genetic material since the bottleneck. For the YAJ population it cannot be excluded that a high degree of kinship between the samples produced the results. The 20 samples of this population could only be collected at the border of, what is expected to be, a much larger population and include two adults and 18 juveniles. It is recommended to either exclude the population from species-focused analyses, or to recollect a better representation of the population.

We cannot easily label the observed genetic diversity (Table 2) to be healthy, high or low, as there is no related, non-threatened *Magnolia* species studied for comparison (Spielman et al. 2004). However, comparisons of the population statistics between the studied threatened species can be made. Firstly, when comparing the statistics of the taxon-datasets, the two populations of *M. hamorii* from the Dominican Republic show a high mean number of alleles (A), in the same extent as the three populations suspected to experience the Wahlund effect. They also have the highest reported values of H_o_ and H_e_ compared with the other Magnolias of this dataset. In the *Splendentes*-normalized-dataset (dataset 3), the statistics of *M. hamorii* do not stand out anymore. However, they remain in the higher range of values, now similar to the statistics found for *M. cubensis*, *M. portoricensis* and *M. splendens*. The latter three species also show A- and H-values in the higher range of values in the calculations of their full taxon-datasets.

Secondly, GRA, MAN and ROD report the three lowest A values in their taxon-datasets, and MAN and ROD show lower A and H values than the GRA and BAR populations, respectively. The lower statistics of the GRA and MAN populations confirm that conservation management of Magnolias in the last remaining forests of Haiti is urgent. Interestingly, even though MAN appeared deforested in an equal, or even higher extent than the ROD population, its alleles tested to be independently associated. LD decreases after recombination events at a rate that depends on the recombination frequency and generally takes more than one generation of random mating to restore, even for (physically) unlinked loci (Slatkin 2008). Hence, the combination of highly disturbed forest and independently associated alleles indicates successful pollination events and surviving new recruits for the MAN population.

Thirdly, the population inbreeding coefficients (F_IS_) of the 14 populations not suspected to be under the Wahlund effect, do not significantly differ from zero. Taking the reproduction biology of Magnolias into consideration, both arguments in favour and against this result can be listed. No (apparent) inbreeding seems likely given that (1) *Magnolia* flowers are reported to be protogynous (Gibbs et al. 1977; Gottsberger 1977; Thien 1974); (2) trees have characteristics that promote outcrossing (Petit and Hampe 2006); and (3) high outcrossing rates have been found in other *Magnolia* species (Tamaki et al. 2009). However, (some degree of) inbreeding was expected given that (1) geitonogamy is theoretically possible (Gibbs et al. 1977; Ishida et al. 2003) provided that they express asynchronous flowering and no self-incompatibility mechanisms; (2) the species are classified as threatened due to small population sizes, high disturbance, and small estimations of extent of occurrence (Rivers et al. 2016); and (3) significant inbreeding has been reported for other Magnolias (Kikuchi and Isagi 2002; Sun et al. 2011). It is possible that recent inbreeding remains undetected due to a time-lag (Kramer et al. 2008; Lugo et al. 1981).

In conclusion, the data showed structuring on three different levels. Firstly, the supraspecific structuring confirms high species integrity with no extensive gene flow between species. Secondly, species sets within islands express lower genetic structuring but no signs of current gene flow, which is interpreted as a more recent shared ancestry. Thirdly, the populations within species also show moderate to strong differentiation, uncorrelated with the distance between the population pairs. The generalisation of extensive gene flow in trees does not withhold in the studied species. Our data support the hypothesis that the generalized concept of extensive gene flow in trees mainly applies to wind pollinated trees or trees that have larger animal vectors such as mammals (Dick et al. 2008). In contrast to the strong structuring, there is no sign of inbreeding, indicating ample gene flow within populations and mechanisms favouring outcrossing. Hence, the reproductive biology of the Neotropical Magnolias appears resilient yet limited in their animal mediated dispersal. A fragmented landscape is expected to strengthen this limitation. Hence, in terms of forest conservation, maintenance of – or preferably: an increase of – connectivity between forest patches would be the most effective strategy to ensure the survival of the species. To practically outline and further investigate the forest connectivity for Magnoliaceae, *Magnolia* SSR research would benefit from studying (1) the reproductive biology of the *Magnolia* trees (pollinators, seed dispersers and phenology) and its limits, shaping the high genetic differentiation between, and high gene flow within populations; (2) the genetic diversity of closely related non-threatened *Magnolia* species, either in fragmented or continuous landscapes, placing past and future SSR *Magnolia* studies on threatened populations in perspective; and (3) splitting *Magnolia* conservation genetic studies according to age, to exclude this potential time-lag and detect whether or not the younger generation of *Magnolia* trees are genetically depauperate (e.g. Graignic et al. 2016; Watanabe et al. 2017).

### Data archiving

Data available from Dryad: 10.5061/dryad.0m625h4.

Genbank accession numbers for the 63 original sequences on which the primers were developed range from MH923371 to MH923433.

## Electronic supplementary material

Supplementary Table S1

Supplementary Table S2

Supplementary Table S3

Supplementary Table S4

Supplementary Figure S5

Supplementary Figure S6

Supplementary Figure S7
