# Supplementary material for: Genetic patterns in Neotropical Magnolias (Magnoliaceae) using de novo developed microsatellite markers
Source: Heredity (Edinb). 2018 Oct 27;122(4):485–500. doi: 10.1038/s41437-018-0151-5 (PMC6460770; doi:10.1038/s41437-018-0151-5)
Supplement: Supplementary file 1 — Supplementary Table S1 [file 41437_2018_151_MOESM1_ESM.doc]

**Supplementary Table S1** Amplification tests. The 17 (sub)species are abbreviated cf. Table 1. The results of the amplification tests are coded: **0** means no amplification; **1** means a single band on the agarose gel; **2** means multiple bands on the agarose gel. The sum of the number of (sub)species (**S**) for which 0, 1, and 2 are coded are given in **S0**, **S1** and **S2**, respectively. The sum of the number of markers (**M**) of which 0, 1, and 2 are coded are given in **M0**, **M1** and **M2**, respectively.

|  | **ACU** | **CRI** | **CUB** | **DEA** | **DOD** | **DOM** | **EKM** | **HAM** | **LAC** | **MAY** | **MIN** | **OBL** | **ORB** | **PAL** | **POR** | **SPL** | **VIR** | **S0** | **S1** | **S2** |
| --- | --- | --- | --- | --- | --- | --- | --- | --- | --- | --- | --- | --- | --- | --- | --- | --- | --- | --- | --- | --- |
| **MA39_023** | 1 | 1 | 0 | 0 | 1 | 1 | 1 | 1 | 1 | 0 | 1 | 0 | 1 | 1 | 1 | 1 | 0 | 5 | 12 | 0 |
| **MA39_046** | 1 | 1 | 1 | 1 | 2 | 1 | 1 | 1 | 1 | 0 | 0 | 0 | 0 | 1 | 0 | 1 | 0 | 6 | 10 | 1 |
| **MA39_142** | 1 | 1 | 2 | 2 | 0 | 1 | 1 | 1 | 1 | 1 | 2 | 2 | 1 | 1 | 1 | 1 | 2 | 1 | 11 | 5 |
| **MA39_159** | 1 | 1 | 1 | 1 | 2 | 1 | 1 | 1 | 1 | 1 | 1 | 1 | 1 | 1 | 1 | 1 | 1 | 0 | 16 | 1 |
| **MA39_165** | 1 | 1 | 0 | 1 | 0 | 1 | 1 | 1 | 2 | 2 | 1 | 2 | 1 | 1 | 0 | 0 | 1 | 4 | 10 | 3 |
| **MA39_182** | 1 | 0 | 1 | 2 | 0 | 1 | 1 | 2 | 1 | 0 | 1 | 1 | 1 | 1 | 2 | 2 | 2 | 3 | 9 | 5 |
| **MA39_185** | 1 | 1 | 0 | 1 | 2 | 1 | 1 | 1 | 1 | 0 | 1 | 1 | 1 | 1 | 1 | 1 | 1 | 2 | 14 | 1 |
| **MA39_199** | 1 | 2 | 1 | 1 | 2 | 1 | 1 | 1 | 1 | 1 | 1 | 1 | 1 | 1 | 1 | 1 | 1 | 0 | 15 | 2 |
| **MA39_236** | 1 | 2 | 1 | 2 | 1 | 2 | 1 | 1 | 1 | 1 | 1 | 1 | 1 | 1 | 1 | 2 | 1 | 0 | 13 | 4 |
| **MA39_259** | 1 | 1 | 2 | 0 | 0 | 1 | 1 | 1 | 2 | 1 | 2 | 2 | 0 | 2 | 1 | 1 | 2 | 3 | 8 | 6 |
| **MA39_263** | 1 | 1 | 2 | 2 | 2 | 2 | 1 | 2 | 1 | 2 | 1 | 1 | 1 | 1 | 1 | 1 | 0 | 1 | 10 | 6 |
| **MA39_287** | 1 | 1 | 2 | 2 | 2 | 1 | 1 | 1 | 1 | 1 | 1 | 1 | 1 | 1 | 1 | 1 | 1 | 0 | 14 | 3 |
| **MA39_327** | 1 | 2 | 2 | 2 | 2 | 1 | 2 | 1 | 1 | 1 | 2 | 2 | 2 | 1 | 2 | 2 | 2 | 0 | 6 | 11 |
| **MA39_342** | 1 | 1 | 2 | 1 | 0 | 1 | 1 | 1 | 1 | 1 | 1 | 1 | 1 | 1 | 1 | 1 | 1 | 1 | 15 | 1 |
| **MA39_348** | 1 | 1 | 1 | 2 | 0 | 1 | 1 | 1 | 1 | 1 | 1 | 1 | 1 | 1 | 1 | 1 | 1 | 1 | 15 | 1 |
| **MA39_442** | 1 | 1 | 1 | 2 | 1 | 1 | 1 | 1 | 1 | 1 | 1 | 1 | 1 | 1 | 1 | 1 | 1 | 0 | 16 | 1 |
| **MA40_045** | 1 | 1 | 0 | 1 | 2 | 1 | 1 | 1 | 1 | 1 | 1 | 1 | 1 | 1 | 1 | 1 | 1 | 1 | 15 | 1 |
| **MA40_072** | 1 | 1 | 1 | 2 | 1 | 1 | 1 | 1 | 1 | 1 | 1 | 1 | 1 | 1 | 2 | 2 | 1 | 0 | 14 | 3 |
| **MA40_136** | 1 | 1 | 1 | 1 | 0 | 1 | 1 | 1 | 1 | 1 | 1 | 1 | 1 | 1 | 1 | 1 | 1 | 1 | 16 | 0 |
| **MA40_175** | 1 | 1 | 2 | 1 | 1 | 1 | 1 | 1 | 1 | 1 | 1 | 1 | 1 | 1 | 1 | 1 | 1 | 0 | 16 | 1 |
| **MA40_223** | 1 | 1 | 0 | 1 | 0 | 1 | 1 | 1 | 1 | 1 | 1 | 1 | 1 | 1 | 1 | 1 | 1 | 2 | 15 | 0 |
| **MA40_282** | 1 | 1 | 0 | 1 | 0 | 1 | 1 | 1 | 1 | 1 | 1 | 1 | 1 | 1 | 1 | 1 | 1 | 2 | 15 | 0 |
| **MA41_076** | 1 | 1 | 1 | 1 | 1 | 1 | 1 | 0 | 0 | 1 | 1 | 1 | 1 | 1 | 1 | 1 | 1 | 2 | 15 | 0 |
| **MA41_215** | 1 | 2 | 1 | 1 | 1 | 1 | 1 | 1 | 0 | 1 | 2 | 2 | 1 | 1 | 1 | 1 | 2 | 1 | 12 | 4 |
| **MA41_264** | 1 | 2 | 1 | 1 | 1 | 2 | 1 | 1 | 1 | 1 | 1 | 2 | 1 | 2 | 1 | 1 | 2 | 0 | 12 | 5 |
| **MA41_373** | 1 | 1 | 2 | 1 | 2 | 1 | 1 | 1 | 1 | 1 | 1 | 1 | 1 | 1 | 1 | 1 | 1 | 0 | 15 | 2 |
| **MA42_001** | 1 | 1 | 1 | 0 | 1 | 1 | 1 | 1 | 0 | 0 | 2 | 2 | 2 | 1 | 1 | 1 | 2 | 3 | 10 | 4 |
| **MA42_028** | 1 | 2 | 1 | 1 | 2 | 2 | 1 | 2 | 1 | 1 | 1 | 1 | 1 | 2 | 2 | 2 | 2 | 0 | 9 | 8 |
| **MA42_059** | 1 | 1 | 0 | 2 | 1 | 1 | 1 | 1 | 2 | 2 | 1 | 1 | 1 | 1 | 1 | 1 | 1 | 1 | 13 | 3 |
| **MA42_063** | 1 | 1 | 1 | 0 | 1 | 1 | 1 | 1 | 0 | 0 | 4 | 4 | 4 | 1 | 1 | 1 | 1 | 3 | 11 | 0 |
| **MA42_072** | 1 | 1 | 1 | 2 | 2 | 1 | 1 | 1 | 2 | 1 | 1 | 1 | 1 | 1 | 1 | 1 | 1 | 0 | 14 | 3 |
| **MA42_077** | 1 | 1 | 1 | 2 | 2 | 1 | 1 | 1 | 1 | 2 | 2 | 2 | 1 | 1 | 1 | 1 | 1 | 0 | 12 | 5 |
| **MA42_083** | 1 | 1 | 1 | 1 | 2 | 1 | 1 | 1 | 2 | 1 | 1 | 0 | 0 | 2 | 2 | 2 | 1 | 2 | 10 | 5 |
| **MA42_087** | 1 | 1 | 2 | 1 | 1 | 1 | 1 | 1 | 1 | 1 | 1 | 1 | 1 | 1 | 1 | 1 | 1 | 0 | 16 | 1 |
| **MA42_102** | 1 | 1 | 0 | 1 | 1 | 1 | 1 | 1 | 1 | 1 | 1 | 1 | 2 | 1 | 1 | 1 | 1 | 1 | 15 | 1 |
| **MA42_126** | 2 | 1 | 1 | 2 | 1 | 1 | 1 | 1 | 2 | 2 | 0 | 0 | 2 | 1 | 1 | 1 | 2 | 2 | 9 | 6 |
| **MA42_147** | 1 | 1 | 1 | 1 | 1 | 1 | 1 | 1 | 0 | 1 | 0 | 0 | 0 | 1 | 1 | 1 | 1 | 4 | 13 | 0 |
| **MA42_166** | 1 | 2 | 0 | 1 | 2 | 1 | 1 | 1 | 0 | 1 | 1 | 2 | 1 | 2 | 1 | 1 | 2 | 2 | 10 | 5 |
| **MA42_185** | 2 | 1 | 1 | 2 | 2 | 2 | 1 | 1 | 2 | 4 | 2 | 2 | 1 | 2 | 1 | 2 | 1 | 0 | 7 | 9 |
| **MA42_197** | 1 | 2 | 1 | 1 | 1 | 2 | 2 | 2 | 0 | 2 | 2 | 0 | 2 | 1 | 2 | 2 | 2 | 2 | 5 | 10 |
| **MA42_202** | 1 | 2 | 1 | 1 | 2 | 1 | 1 | 1 | 2 | 2 | 1 | 2 | 1 | 2 | 1 | 2 | 2 | 0 | 9 | 8 |
| **MA42_203** | 1 | 1 | 1 | 2 | 2 | 1 | 1 | 1 | 2 | 0 | 2 | 2 | 0 | 1 | 1 | 1 | 2 | 2 | 9 | 6 |
| **MA42_231** | 1 | 1 | 1 | 1 | 1 | 1 | 1 | 1 | 1 | 1 | 1 | 1 | 1 | 1 | 1 | 1 | 1 | 0 | 17 | 0 |
| **MA42_241** | 1 | 1 | 2 | 1 | 0 | 1 | 1 | 1 | 1 | 1 | 1 | 1 | 1 | 1 | 1 | 1 | 1 | 1 | 15 | 1 |
| **MA42_247** | 1 | 1 | 0 | 1 | 0 | 1 | 1 | 1 | 2 | 1 | 1 | 2 | 1 | 1 | 1 | 1 | 2 | 2 | 12 | 3 |
| **MA42_253** | 1 | 1 | 1 | 1 | 1 | 2 | 1 | 2 | 1 | 1 | 1 | 1 | 2 | 1 | 2 | 2 | 1 | 0 | 12 | 5 |
| **MA42_255** | 1 | 1 | 2 | 1 | 2 | 1 | 1 | 1 | 1 | 1 | 1 | 1 | 1 | 1 | 1 | 1 | 1 | 0 | 15 | 2 |
| **MA42_265** | 1 | 1 | 1 | 1 | 1 | 1 | 1 | 1 | 1 | 1 | 1 | 1 | 1 | 1 | 1 | 1 | 1 | 0 | 17 | 0 |
| **MA42_274** | 1 | 2 | 1 | 1 | 1 | 2 | 2 | 2 | 1 | 1 | 2 | 2 | 2 | 2 | 2 | 2 | 2 | 0 | 6 | 11 |
| **MA42_279** | 1 | 1 | 1 | 1 | 1 | 1 | 1 | 1 | 1 | 1 | 1 | 1 | 1 | 1 | 1 | 1 | 1 | 0 | 17 | 0 |
| **MA42_293** | 2 | 2 | 2 | 2 | 0 | 2 | 2 | 2 | 2 | 2 | 2 | 2 | 2 | 2 | 2 | 2 | 2 | 1 | 0 | 16 |
| **MA42_296** | 1 | 1 | 1 | 2 | 1 | 1 | 1 | 1 | 1 | 1 | 1 | 2 | 1 | 1 | 1 | 1 | 1 | 0 | 15 | 2 |
| **MA42_333** | 1 | 1 | 2 | 1 | 2 | 1 | 1 | 0 | 0 | 0 | 0 | 1 | 0 | 1 | 1 | 1 | 1 | 5 | 10 | 2 |
| **MA42_334** | 2 | 2 | 1 | 1 | 0 | 1 | 1 | 1 | 1 | 1 | 1 | 1 | 1 | 1 | 1 | 1 | 1 | 1 | 14 | 2 |
| **MA42_372** | 1 | 1 | 2 | 1 | 2 | 1 | 1 | 1 | 1 | 1 | 1 | 1 | 1 | 1 | 1 | 1 | 1 | 0 | 15 | 2 |
| **MA42_397** | 1 | 1 | 1 | 1 | 1 | 1 | 1 | 1 | 2 | 1 | 1 | 2 | 1 | 1 | 1 | 1 | 1 | 0 | 15 | 2 |
| **MA42_413** | 1 | 1 | 1 | 2 | 1 | 1 | 1 | 1 | 2 | 1 | 0 | 0 | 0 | 1 | 1 | 1 | 1 | 3 | 12 | 2 |
| **MA42_421** | 1 | 1 | 0 | 1 | 0 | 1 | 1 | 1 | 1 | 1 | 1 | 2 | 1 | 1 | 1 | 1 | 1 | 2 | 14 | 1 |
| **MA42_471** | 1 | 2 | 1 | 0 | 1 | 2 | 1 | 1 | 0 | 0 | 1 | 1 | 2 | 1 | 1 | 1 | 1 | 3 | 11 | 3 |
| **MA42_472** | 1 | 1 | 1 | 2 | 1 | 1 | 1 | 1 | 2 | 2 | 1 | 0 | 1 | 1 | 1 | 1 | 1 | 1 | 13 | 3 |
| **MA42_481** | 1 | 1 | 0 | 1 | 1 | 1 | 1 | 1 | 1 | 1 | 2 | 0 | 0 | 1 | 1 | 1 | 1 | 3 | 13 | 1 |
| **MA42_491** | 1 | 1 | 1 | 0 | 1 | 1 | 1 | 0 | 0 | 0 | 0 | 1 | 0 | 1 | 1 | 0 | 1 | 7 | 10 | 0 |
| **MA42_495** | 1 | 1 | 1 | 1 | 1 | 1 | 1 | 1 | 1 | 1 | 1 | 1 | 1 | 1 | 1 | 1 | 1 | 0 | 17 | 0 |
| **M0** | 0 | 1 | 3 | 6 | 6 | 0 | 0 | 3 | 10 | 10 | 6 | 9 | 9 | 0 | 2 | 2 | 3 |  |  |  |
| **M1** | 59 | 49 | 56 | 38 | 43 | 53 | 59 | 53 | 39 | 43 | 44 | 35 | 44 | 54 | 52 | 49 | 44 |  |  |  |
| **M2** | 4 | 13 | 4 | 19 | 14 | 10 | 4 | 7 | 14 | 9 | 12 | 18 | 9 | 9 | 9 | 12 | 16 |  |  |  |
