# Supplementary material for: Genetic patterns in Neotropical Magnolias (Magnoliaceae) using de novo developed microsatellite markers
Source: Heredity (Edinb). 2018 Oct 27;122(4):485–500. doi: 10.1038/s41437-018-0151-5 (PMC6460770; doi:10.1038/s41437-018-0151-5)
Supplement: Supplementary file 2 — Supplementary Table S2 [file 41437_2018_151_MOESM2_ESM.doc]

**Supplementary Table S2** Polymorphism tests. The 10 taxa are abbreviated cf. Table 1. The results of the polymorphism tests are coded: **A**: polymorphic and unambiguous; **B**: monomorphic genotyped for all 20 individuals; **C**: monomorphic genotyped for 4 or 8 individuals; **D**: ambiguous to score; **E**: not amplifying (well) in the PCR. The sum of the number of (sub)species (**S**) for which A, B, C, D and E are coded are given in **SA**, **SB**, **SC**, **SD** and **SE**, respectively. The sum of the number of markers (**M**) of which A, B, C, D and E are coded are given in **MA**, **MB**, **MC**, **MD** and **ME**, respectively. Some marker × species combinations were not submitted to fragment analyses due to no amplification or double amplification products in the amplification tests (0 and 2, respectively, in Supplementary Table S1). The number of (sub)species submitted to fragment analyses per marker is given in **SF** (maximum = 10). The number of markers tested in fragment analyses per (sub)species is given in **MF** (maximum = 63). The markers employed in the taxon-datasets are those coded with an A for the taxon at hand. The ten markers employed in the *Splendentes*-normalized dataset (dataset 3) are the markers labelled with an asterisk.

|  | **ACU** | **CUB** | **DOD** | **DOM** | **EKM** | **HAM** | **LAC** | **PAL** | **POR** | **SPL** | **SA** | **SB** | **SC** | **SD** | **SE** | **SF** | **SA+B+C** |
| --- | --- | --- | --- | --- | --- | --- | --- | --- | --- | --- | --- | --- | --- | --- | --- | --- | --- |
| **MA39_023** | A | A | A | A | A | A | A | A | A | A | 10 | 0 | 0 | 0 | 0 | 10 | 10 |
| **MA39_046** | B | A |  | E | C | E | D | E | E | E | 1 | 1 | 1 | 1 | 5 | 9 | 3 |
| **MA39_142** | C | C | C | C | C | C | A | C | A | C | 2 | 0 | 8 | 0 | 0 | 10 | 10 |
| **MA39_159** | E | E | A | E | D | E | A | C | E | E | 2 | 0 | 1 | 1 | 6 | 10 | 3 |
| **MA39_165** | C | C |  | A | C | C | D | D | E | C | 1 | 0 | 5 | 2 | 1 | 9 | 6 |
| **MA39_182** | E | E | A | E | D | C | A | E | E |  | 2 | 0 | 1 | 1 | 5 | 9 | 3 |
| **MA39_185*** | A | A | A | A | A | A | A | A | A | A | 10 | 0 | 0 | 0 | 0 | 10 | 10 |
| **MA39_199** | A | A | A | A | C | A | C | A | A | C | 7 | 0 | 3 | 0 | 0 | 10 | 10 |
| **MA39_236** | E | E | D | E | A | A | A | E | A | E | 4 | 0 | 0 | 1 | 5 | 10 | 4 |
| **MA39_259** | C | C | A | C | A | E | A | A | C | C | 4 | 0 | 5 | 0 | 1 | 10 | 9 |
| **MA39_263** | A | B |  |  | A | C | C | C | C | C | 2 | 1 | 5 | 0 | 0 | 8 | 8 |
| **MA39_287** | C | C | A | C | C | C | A | C | C | C | 2 | 0 | 8 | 0 | 0 | 10 | 10 |
| **MA39_327** | E | E |  | E |  | E | A | E |  |  | 1 | 0 | 0 | 0 | 5 | 6 | 1 |
| **MA39_342** | E | E |  | E | E | E | A | E | E | E | 1 | 0 | 0 | 0 | 8 | 9 | 1 |
| **MA39_348** | E | E | E | E | E | E | A | E | A | A | 3 | 0 | 0 | 0 | 7 | 10 | 3 |
| **MA39_442** | A | A | A | C | C | C | A | C | B | C | 4 | 1 | 5 | 0 | 0 | 10 | 10 |
| **MA40_045** | A | A | B | D | B | A | B | A | A | D | 5 | 3 | 0 | 2 | 0 | 10 | 8 |
| **MA40_072** | C | C | E | D | A | C | A | D |  |  | 2 | 0 | 3 | 2 | 1 | 8 | 5 |
| **MA40_136** | C | C | A | C | C | C | C | C | C | A | 2 | 0 | 8 | 0 | 0 | 10 | 10 |
| **MA40_175** | C | C | B | C | C | C | C | C | C | A | 1 | 1 | 8 | 0 | 0 | 10 | 10 |
| **MA40_223** | C | C | C | D | C | A | C | C | C | A | 2 | 0 | 7 | 1 | 0 | 10 | 9 |
| **MA40_282*** | A | A | A | A | A | A | A | A | A | A | 10 | 0 | 0 | 0 | 0 | 10 | 10 |
| **MA41_076** | A | A | D | E | A | E | A | C | A | A | 6 | 0 | 1 | 1 | 2 | 10 | 7 |
| **MA41_215** | D | D |  | D | A | A |  | D | A | D | 3 | 0 | 0 | 5 | 0 | 8 | 3 |
| **MA41_264** | A | A | D |  | A | E | D |  | E | E | 3 | 0 | 0 | 2 | 3 | 8 | 3 |
| **MA41_373*** | A | A | B | A | A | A | A | A | A | A | 9 | 1 | 0 | 0 | 0 | 10 | 10 |
| **MA42_001*** | A | A | E | A | A | A | E | A | A | A | 8 | 0 | 0 | 0 | 2 | 10 | 8 |
| **MA42_028** | A | A | E |  | A |  | A |  |  |  | 4 | 0 | 0 | 0 | 1 | 5 | 4 |
| **MA42_059** | C | C | E | A | C | C | E | A | C | C | 2 | 0 | 6 | 0 | 2 | 10 | 8 |
| **MA42_063** | A | A | E | C | E | C | E | D | A | A | 4 | 0 | 2 | 1 | 3 | 10 | 6 |
| **MA42_072** | C | C | A | C | C | C | D | C | C | E | 1 | 0 | 7 | 1 | 1 | 10 | 8 |
| **MA42_077*** | B | A | A | A | A | A | E | A | A | A | 8 | 1 | 0 | 0 | 1 | 10 | 9 |
| **MA42_083** | A | A | D | C | A | A | D | A | E | D | 5 | 0 | 1 | 3 | 1 | 10 | 6 |
| **MA42_087** | A | A | C | D | A | E | C | E | A | E | 4 | 0 | 2 | 1 | 3 | 10 | 6 |
| **MA42_102** | D | D |  | D | A | A | D | D | A | A | 4 | 0 | 0 | 5 | 0 | 9 | 4 |
| **MA42_126** | A | D | E | A | B | A | E | E | A | A | 5 | 1 | 0 | 1 | 3 | 10 | 6 |
| **MA42_147** | D | D | E | C | C | C | D | C | A | A | 2 | 0 | 4 | 3 | 1 | 10 | 6 |
| **MA42_166** | A | A | D | A | D | D |  |  | D | D | 3 | 0 | 0 | 5 | 0 | 8 | 3 |
| **MA42_185** |  |  | D |  | D | D | E |  | A |  | 1 | 0 | 0 | 3 | 1 | 5 | 1 |
| **MA42_197** | D | A |  |  |  |  |  | D |  |  | 1 | 0 | 0 | 2 | 0 | 3 | 1 |
| **MA42_202** | A | D | D | D | A | D | D | D | D |  | 2 | 0 | 0 | 7 | 0 | 9 | 2 |
| **MA42_203*** | A | A | E | A | A | A | E | A | A | A | 8 | 0 | 0 | 0 | 2 | 10 | 8 |
| **MA42_231*** | A | A | A | A | A | A | A | A | A | A | 10 | 0 | 0 | 0 | 0 | 10 | 10 |
| **MA42_241** | A | A | C | D | E | A | C | A | D | A | 5 | 0 | 2 | 2 | 1 | 10 | 7 |
| **MA42_247** | A | A | E | D | A | D | C | D | D | D | 3 | 0 | 1 | 5 | 1 | 10 | 4 |
| **MA42_253** | C | C | C |  | A |  | E | D |  |  | 1 | 0 | 3 | 1 | 1 | 6 | 4 |
| **MA42_255*** | A | A | A | A | A | A | A | A | A | A | 10 | 0 | 0 | 0 | 0 | 10 | 10 |
| **MA42_265** | A | B | D | C | C | C | C | C | C | C | 1 | 1 | 7 | 1 | 0 | 10 | 9 |
| **MA42_274** | A | A | A |  |  |  | A |  |  |  | 4 | 0 | 0 | 0 | 0 | 4 | 4 |
| **MA42_279** | A | D | E | E | D | C | E | D | E | E | 1 | 0 | 1 | 3 | 5 | 10 | 2 |
| **MA42_293** |  |  |  | A | C | D | E | A | E | D | 2 | 0 | 1 | 2 | 2 | 7 | 3 |
| **MA42_296** | A | A | D | D | D | A | E | D | D | D | 3 | 0 | 0 | 6 | 1 | 10 | 3 |
| **MA42_333** | D | D | A | D | D | D |  | D | D | E | 1 | 0 | 0 | 7 | 1 | 9 | 1 |
| **MA42_334** | D | D | D | A | D | D | C | D | D | E | 1 | 0 | 1 | 7 | 1 | 10 | 2 |
| **MA42_372** | D | D | A | D | D | D | D | D | C | C | 1 | 0 | 2 | 7 | 0 | 10 | 3 |
| **MA42_397*** | A | A | A | A | A | A | D | A | A | A | 9 | 0 | 0 | 1 | 0 | 10 | 9 |
| **MA42_413** | B | A | E | D | A | A | E | D | A | A | 5 | 1 | 0 | 2 | 2 | 10 | 6 |
| **MA42_421*** | A | A | A | A | A | B | A | A | A | A | 9 | 1 | 0 | 0 | 0 | 10 | 10 |
| **MA42_471** | A | A | A | D | B | A | A | A | A | A | 8 | 1 | 0 | 1 | 0 | 10 | 9 |
| **MA42_472** | A | A | E | A | A | A | E | A | A | A | 8 | 0 | 0 | 0 | 2 | 10 | 8 |
| **MA42_481** | A | A | E | A | A | A | E | E | A | A | 7 | 0 | 0 | 0 | 3 | 10 | 7 |
| **MA42_491** | D | D | E | D | A | D | E | E | E |  | 1 | 0 | 0 | 4 | 4 | 9 | 1 |
| **MA42_495** | D | D | A | A | A | D | A | E | A | C | 5 | 0 | 1 | 3 | 1 | 10 | 6 |
| **MA** | **32** | **31** | **21** | **21** | **30** | **24** | **23** | **20** | **29** | **25** |  |  |  |  |  |  |  |
| **MB** | 3 | 2 | 3 | 0 | 3 | 1 | 1 | 0 | 1 | 0 |  |  |  |  |  |  |  |
| **MC** | 11 | 11 | 5 | 11 | 14 | 15 | 10 | 12 | 10 | 11 |  |  |  |  |  |  |  |
| **MD** | 9 | 11 | 10 | 15 | 9 | 10 | 10 | 15 | 7 | 7 |  |  |  |  |  |  |  |
| **ME** | 6 | 6 | 15 | 9 | 4 | 9 | 15 | 11 | 10 | 10 |  |  |  |  |  |  |  |
| **MF** | 61 | 61 | 54 | 56 | 60 | 59 | 59 | 58 | 57 | 53 |  |  |  |  |  |  |  |
| **MA+MB+MC** | 46 | 44 | 29 | 32 | 47 | 40 | 34 | 32 | 40 | 36 |  |  |  |  |  |  |  |
