# Supplementary material for: Genetic patterns in Neotropical Magnolias (Magnoliaceae) using de novo developed microsatellite markers
Source: Heredity (Edinb). 2018 Oct 27;122(4):485–500. doi: 10.1038/s41437-018-0151-5 (PMC6460770; doi:10.1038/s41437-018-0151-5)
Supplement: Supplementary file 3 — Supplementary Table S3 [file 41437_2018_151_MOESM3_ESM.doc]

**Supplementary Table S3** Microsatellite primer information. Primers that scored polymorphic, unambiguous and with no null-alleles for at least one of the ten tested species are listed. Primers with the locus name starting with **MA39** are developed on *Magnolia lacandonica*, **MA40** on *M. mayae*, **MA41** on *M. dealbata* and **MA42** on *M. cubensis* subsp. *acunae*. All SSR primers run with an annealing temperature (T_a_) of 60°C. Size range of all the amplicons for the specific primer pair are given applicable to all the species on which the primer pair was tested to be valuable. The original size of the fragment on which the primer pair is developed is given between square brackets. The highlighted primers have an error reported in one of the duplicated genotypes. **NT**: not tested.

| Locus Name | Primer sequences (5’-3’) | Repeat motif | Size range  [size] (bp) | Error rate (%) [#errors/#tests] | GenBank Accession Number |
| --- | --- | --- | --- | --- | --- |
| MA39_023 | F: ATCACGCATCTGCACAGACA R: GGACAACGAACGTCTGGCTA | (AG)7 | 118–198 [97] | 0% [0/120] | MH923371 |
| MA39_046 | F: CCATCCAGAGCACGAGTGTT R: CACACGGAAACTCCAGACCA | (AG)18 | 132–134 [137] | 0% [0/4] | MH923372 |
| MA39_142 | F: ATGTGGCCTACGTTGCTCAA R: GGATCTCAGACCCATCGTGC | (TAA)10 | 207–221 [190] | 0% [0/48] | MH923373 |
| MA39_159 | F: ATCAGGAGTGTAACGCCACC R: GGCGAGCTCGTTAGATCCTC | (TC)16 | 146–178 [139] | 0% [0/67] | MH923374 |
| MA39_165 | F: AATGTAGTGGGTCCGGCTTC R: CCAAACCATGTGCGTCCTTG | (TC)18 | 197–199 [181] | 0% [0/12] | MH923375 |
| MA39_182 | F: CTACACGGGTGAAGCCTACC R: GGCCGTAATCAGAGTCCACC | (TC)12 | 144–148 [129] | 0% [0/33] | MH923376 |
| MA39_185 | F: CGGGTGTTGTAGATGACGCT R: AAGACACGGAATGGGACGAG | (AG)15 | 231–358 [209] | 0% [0/107] | MH923377 |
| MA39_199 | F: CGCCCACATCTACCTCTTCG R: TCCAGGAGTTTCTGTGCACC | (GGA)5 | 193–222 [187] | 0% [0/45] | MH923378 |
| MA39_236 | F: GGCAGAAGCAAGGAAGAGGA R: GAATCAAACCGCAGCTCGAC | (GA)19 | 163–194 [153] | 0% [0/52] | MH923379 |
| MA39_259 | F: TGATAGAGTGGGATGGCGGA R: TGCTGCTTTGAGGCCTGTTA | (CT)11 | 105–179 [96] | 0% [0/98] | MH923380 |
| MA39_263 | F: GTAGCCATGTGGGTCTGTCC  R: AGTTGGTAGGGCACATGTCC | (CT)13 | 148–158 [126] | 0% [0/12] | MH923381 |
| MA39_287 | F: CCTCGAGCATCACCACCTTC R: GGTGGACCCTACACATGTGG | (AG)16 | 142–176 [129] | 0% [0/68] | MH923382 |
| MA39_327 | F: CCCATTCGCAATCTTACGCC R: TGGTTTCAATGCGAGACGGT | (CT)15 | 136–172 [117] | 3.33% [2/60] | MH923383 |
| MA39_342 | F: TCCCTTCAGTCTTCACACGC R: AAAGGAGCGTTGAGTGGTGG | (TC)14 | 164–208 [146] | 0% [0/24] | MH923384 |
| MA39_348 | F: GTAGAGCTCCCATGCCTCAC R: GGGCTGTCTACTGGATGGAC | (TC)17 | 150–180 [119] | 0% [0/28] | MH923385 |
| MA39_442 | F: AGTCGATCCTCTTGCTGCAC R: GAGGGAGCATCGGCCATTAC | (AAG)8 | 133–145 [109] | 0% [0/53] | MH923386 |
| MA40_045 | F: TTGTGGGCCAAGCTCGATAG R: ATTGTGGCATGTACCTCGCA | (TC)13 | 246–292 [231] | 1.10% [1/91] | MH923387 |
| MA40_072 | F: ATCCGATTCCCATTCGGACG R: CTGCCGGAGAAGAGAACGAG | (CT)14 | 117–137 [111] | 3.85% [1/26] | MH923388 |
| MA40_136 | F: CTGGGCATTGCAGAGTAGCT R: CATCCCAGCAGTTACGACGA | (GCC)6 | 116–128 [108] | 0% [0/21] | MH923389 |
| MA40_175 | F: CGTTCTGCGCGATCAATCTC R: GCATCCGAATCCCAGCTACA | (GCT)6 | 105–111 [91] | 0% [0/14] | MH923390 |
| MA40_223 | F: TTCAGTGGCTGGAGCTTCAG R: GGAGCATCTTGGCCTTTGGA | (GAT)5 | 116–132 [93] | 0% [0/21] | MH923391 |
| MA40_282 | F: TCTCTTTCCCTCCGTCCTCC R: TCTTCCGGCTTCATGTCGTC | (GA)15 | 128–166 [116] | 0% [0/66] | MH923392 |
| MA41_076 | F: AACAACGCTGGGTGATGGAA R: TGGAGTTGACGCCTCTAGGA | (GA)26 | 169–209 [176] | 1.59% [1/63] | MH923393 |
| MA41_215 | F: TTCAGCCAACTGGAATCCGG R: GTGCCTTGAAATGAGCTGGC | (AG)18 | 219–237 [207] | 0% [0/43] | MH923394 |
| MA41_264 | F: AACAGCCTTTGGGAAGTGCA R: CAGCCATTCCGCTTCCCTTA | (GA)15 | 236–245 [173] | 0% [0/28] | MH923395 |
| MA41_373 | F: GCGCCCAATCAGAACACAAC R: GGGAAGAGCTTCTTTCGCCA | (CT)16 | 166–207 [165] | 0% [0/94] | MH923396 |
| MA42_001 | F: ATCCGACCCAACATGGTGAC R: AGCCGAGTCTGAGCTGAGTA | (TC)11 | 144–169 [130] | 0% [0/82] | MH923397 |
| MA42_028 | F: GGATCGTCTTCCGCCATTCT  R: TTCCGTACGATGCTCCCATG | (CT)33 | 129–147 [151] | 0% [0/31] | MH923398 |
| MA42_059 | F: AGGGACTCGGCATCTATGGA R: GAGTCGACTCAGCAACTCCC | (AG)8 | 246–248 [217] | 0% [0/25] | MH923399 |
| MA42_063 | F: ATAGCAACAACGTAGCCGGT R: TGGCGAGGTCCCTCTACTAC | (GA)14 | 218–250 [203] | 0% [0/35] | MH923400 |
| MA42_072 | F: CCCACCTAGGTTTCCAGTGC R: TGCGTTCGAAAGGCACAATG | (CA)5 | 269–273 [245] | 0% [0/8] | MH923401 |
| MA42_077 | F: GAGACATGGAACCCACACGT R: CTGGTGGTCTAGCCGATCTG | (AG)8 | 234–284 [211] | 0% [0/99] | MH923402 |
| MA42_083 | F: GTCTTCCACGGGAGCAAGAG R: CGAGTTGGACCCAGTGAGTC | (GAA)17 | 101–145 [120] | 0% [0/47] | MH923403 |
| MA42_087 | F: TAAGTCAGAACCCAGCTGGC R: GGCGAATCGGGACCCTTTAA | (GA)17 | 179–204 [157] | 0% [0/18] | MH923404 |
| MA42_102 | F: CTGTCTCAGCGTCTCACTCC R: AGACGAAGGGAGGGAAGGAG | (CT)21 | 89–116 [90] | 0% [0/43] | MH923405 |
| MA42_126 | F: CACATCGTCCGTCCAGACAT R: TCGCCTAGCCAATAGTCTGC | (AT)9 | 126–135 [103] | 0% [0/46] | MH923406 |
| MA42_147 | F: AAATCACGGTCGGGATTCGA R: GGGCATGAGCTGTGGATCTT | (CT)8 | 251–263 [222] | 0% [0/33] | MH923407 |
| MA42_166 | F: CTCTTGGCCGATGGAGATGG R: GGACGTGGGAAGCATCTCTG | (TC)13 | 126–142 [122] | 0% [0/25] | MH923408 |
| MA42_185 | F: CTGCTGGACGGTCTGGATTG  R: TCGAGCTGTCCATCATCACG | (AG)11 | 120–153 [90] | 0% [0/14] | MH923409 |
| MA42_197 | F: GGCTAGCCGACTTAACCTGA  R: CGTCAAGTCTGAGTCGGGTC | (TC)25 | 185–205 [184] | NT | MH923410 |
| MA42_202 | F: AGGGAGGGCTCATAGTGGTG R: CGGACAGTGGTGTGGTTCAT | (CT)11 | 188–220 [122] | 0% [0/16] | MH923411 |
| MA42_203 | F: TGAAGAACACAGGCCATGGA R: GAGAGGTGCTTCACGGGTAG | (TC)16 | 105–136 [102] | 1% [1/100] | MH923412 |
| MA42_231 | F: GGGTGCGAAATGTGCATCAA R: GGGCCAGTGAGCATTAGAGC | (AG)14 | 152–194 [131] | 0% [1/77] | MH923413 |
| MA42_241 | F: GGGTACCCTATGGTCCAACC R: GTCCGACTAAGGCCCATTGT | (CA)11 | 108–114 [92] | 0% [0/30] | MH923414 |
| MA42_247 | F: AGGTGGGCAATCATACAAGGG R: AGGGCCCATAGTACAGGGTT | (AG)24 | 120–154 [112] | 0% [0/23] | MH923415 |
| MA42_253 | F: GACGGACTTAGAGCATGGGT  R: GCTTGAATTTGTGGTGGCCC | (TC)36 | 154–178 [182] | 0% [0/8] | MH923416 |
| MA42_255 | F: ACGTGGGTCGAGGATCAAGT R: GGACCCACCTCCAACAGATC | (AG)14 | 144–174 [137] | 1.89% [2/106] | MH923417 |
| MA42_265 | F: CGCACACCAAAGCTGCATT R: CGGCTACTTCCCAAGGGATG | (AAG)12 | 251–254 [238] | 0% [0/10] | MH923418 |
| MA42_274 | F: CAGCCATTCCTTGAGATGGGT R: GCCGAAACGATCTCTTCCCT | (GA)18 | 161–209 [154] | 0% [0/48] | MH923419 |
| MA42_279 | F: AGACAGTCCAGTAGGGTGGA  R: GAGCTCCTCCAATCTCCACC | (AG)17 | 142–150 [142] | 0% [0/5] | MH923420 |
| MA42_293 | F: TGCAACTGAGACGAGTTGGG R: GGTACGGACTAGGGTACAGGT | (GA)16 | 120–124 [109] | 0% [0/24] | MH923421 |
| MA42_296 | F: TTGACAGTCTGGCAAGGTGG R: GAGGGCTCATAGTGGTGGC | (AG)15 | 166–180 [144] | 0% [0/17] | MH923422 |
| MA42_333 | F: GGAGTCAAGCGACAACTCCA R: GTGTGCATGTGGATAAGCCA | (GA)33 | 225–283 [257] | 0% [0/8] | MH923423 |
| MA42_334 | F: TGCAGATGGTGGCAATGCTT R: GGTCAAGTTTACACCGCGGA | (TCA)10 | 154–172 [143] | 0% [0/14] | MH923424 |
| MA42_372 | F: ATCCGAACTCGACTGTGACT R: CCTACCCAAGTCAGCCCATC | (TC)20 | 145–217 [141] | 0% [0/7] | MH923425 |
| MA42_397 | F: TAGTAGCAGGGTCCCTCCTC R: TCCATTCATTAGGGTGGGCA | (TC)20 | 100–163 [98] | 0% [0/9] | MH923426 |
| MA42_413 | F: GCCGAGTGCAAGCCATAAGG R: TGCACCTAAGCTCCACAGTC | (GA)9 | 127–153 [103] | 0% [0/45] | MH923427 |
| MA42_421 | F: GACAGCAGACCTGACCGATT R: GACCAGTGCATCCCATCAAA | (TC)10 | 298–390 [280] | 0% [0/69] | MH923428 |
| MA42_471 | F: TGATGAAGAGCCCAGATCGTC R: TGGCCTTGTTCTCCATACGT | (GA)16 | 163–230 [153] | 0% [0/134] | MH923429 |
| MA42_472 | F: AGAGTTACACATGCAAACCCG R: TGATGTTGTTGCTCGGCTGA | (AG)17 | 157–205 [140] | 0% [0/97] | MH923430 |
| MA42_481 | F: CGATCTGAGTCCGCAAGAGT R: GACGCAGAAATCTCAGCAAGA | (TC)15 | 212–238 [197] | 0% [0/62] | MH923431 |
| MA42_491 | F: TGGAAGAGTCAACCACACTGG  R: ACTGTAATGGACCAACAGCCA | (CT)27 | 103–123 [108] | 0% [0/8] | MH923432 |
| MA42_495 | F: TGCATCTCCTCATCCTCCCA R: ACGCCATTCAATTACCTACGG | (GA)26 | 92–152 [97] | 0% [0/51] | MH923433 |
