# Supplementary material for: Genetic patterns in Neotropical Magnolias (Magnoliaceae) using de novo developed microsatellite markers
Source: Heredity (Edinb). 2018 Oct 27;122(4):485–500. doi: 10.1038/s41437-018-0151-5 (PMC6460770; doi:10.1038/s41437-018-0151-5)
Supplement: Supplementary file 4 — Supplementary Table S4 [file 41437_2018_151_MOESM4_ESM.doc]

**Supplementary Table S4** Summary statistics given per (sub)species, marker and location. **N**: (mean) number of genotyped individuals, consistent individuals with no peaks: *****. **A**: mean number of alleles. **H_o_**: observed heterozygosity. **H_e_**: expected heterozygosity. **F_IS_**: inbreeding coefficient, significant deviations from Hardy-Weinberg Proportions (HWP): ***** (p = 0.05) and ****** (p = 0.05, Bonferroni corrected), **M**: monomorphic. **A_0_**: estimated null allele frequency, locus-population combinations recognized by MICROCHECKER: *****. Statistics averaged per population: “Pop (number of SSR markers)”. **^1, 2^**: excluded markers. **SD** = Standard Deviation. **a** *Magnolia cubensis* subsp. *acunae*: 32 polymorphic SSR markers. **b** *Magnolia cubensis* subsp. *cubensis*: 31 polymorphic SSR markers. **c** *Magnolia dodecapetala*: 21 polymorphic SSR markers. **d** *Magnolia domingensis*: 21 polymorphic SSR markers. **e** *Magnolia ekmanii*: 30 polymorphic SSR markers. **f** *Magnolia hamorii*: 24 polymorphic SSR markers. **g** *Magnolia lacandonica*: 23 polymorphic SSR markers. **h** *Magnolia pallescens*: 20 polymorphic SSR markers. **i** *Magnolia portoricensis*: 29 polymorphic SSR markers. **j** *Magnolia splendens*: 25 polymorphic SSR markers.

**Supplementary Table S4a**: *Magnolia cubensis* subsp. *acunae*: 32 polymorphic SSR markers.

| **Population** | TOP | | | | | |
| --- | --- | --- | --- | --- | --- | --- |
| **SSR marker** | **N** | **A** | **H_o_** | **H_e_** | **F_is_** | **A_0_** |
| MA39_023 | 20 | 4 | 0.500 | 0.596 | 0.186 | 0.069 |
| MA39_185 | 20 | 10 | 0.750 | 0.816 | 0.107* | 0.004 |
| MA39_199 | 18 | 3 | 0.500 | 0.440 | -0.109 | 0 |
| MA39_263 | 20 | 2 | 0.450 | 0.469 | 0.066 | 0.013 |
| MA39_442 | 20 | 2 | 0.300 | 0.255 | -0.152 | 0 |
| MA40_045 | 20 | 8 | 0.700 | 0.806 | 0.157 | 0.068 |
| MA40_282 | 20 | 9 | 0.850 | 0.835 | 0.008 | 0 |
| MA41_076 | 20 | 2 | 0.050 | 0.049 | 0 | 0 |
| MA41_264 | 20 | 4 | 0.750 | 0.724 | -0.011 | 0 |
| MA41_373 | 20 | 7 | 0.800 | 0.770 | -0.013 | 0 |
| MA42_001 | 20 | 5 | 0.600 | 0.693 | 0.159 | 0.068 |
| MA42_028^1^ | 20 | 6 | 0.300 | 0.518 | 0.441** | 0.161* |
| MA42_063 | 19 | 11 | 0.895 | 0.855 | -0.02 | 0 |
| MA42_083 | 20 | 8 | 0.800 | 0.643 | -0.221 | 0 |
| MA42_087 | 20 | 7 | 0.750 | 0.743 | 0.016 | 0.016 |
| MA42_126 | 20 | 3 | 0.350 | 0.366 | 0.07 | 0.016 |
| MA42_166 | 20 | 4 | 0.600 | 0.469 | -0.256 | 0 |
| MA42_202 | 20 | 11 | 0.750 | 0.643 | -0.142 | 0 |
| MA42_203 | 20 | 6 | 0.850 | 0.793 | -0.047 | 0 |
| MA42_231 | 20 | 3 | 0.350 | 0.486 | 0.304 | 0.092 |
| MA42_241 | 20 | 3 | 0.550 | 0.595 | 0.101 | 0.009 |
| MA42_247 | 20 | 9 | 0.900 | 0.823 | -0.069 | 0 |
| MA42_255 | 20 | 5 | 0.550 | 0.646 | 0.174 | 0.048 |
| MA42_265 | 20 | 2 | 0.100 | 0.095 | -0.027 | 0 |
| MA42_274 | 20 | 5 | 0.550 | 0.445 | -0.212 | 0 |
| MA42_279 | 20 | 5 | 0.600 | 0.623 | 0.062 | 0 |
| MA42_296 | 20 | 7 | 0.700 | 0.649 | -0.053 | 0 |
| MA42_397 | 20 | 5 | 0.550 | 0.654 | 0.184* | 0 |
| MA42_421 | 20 | 8 | 0.800 | 0.779 | -0.002 | 0 |
| MA42_471 | 19 | 3 | 0.526 | 0.543 | 0.058 | 0 |
| MA42_472 | 20 | 5 | 0.500 | 0.586 | 0.172 | 0.030 |
| MA42_481 | 20 | 3 | 0.500 | 0.445 | -0.098 | 0 |
| **Pop (31^1^)** | 19.871 | 5.452 | 0.594 | 0.591 | 0.021 |  |
| **SD (31^1^)** | 0.077 | 0.493 | 0.038 | 0.037 |  |  |

**Supplementary Table S4b**: *Magnolia cubensis* subsp. *cubensis*: 31 polymorphic SSR markers.

| **Population** | PIC | | | | | |
| --- | --- | --- | --- | --- | --- | --- |
| **SSR marker** | **N** | **A** | **H_o_** | **H_e_** | **F_is_** | **A_0_** |
| MA39_023 | 20 | 4 | 0.550 | 0.629 | 0.150 | 0.048 |
| MA39_046 | 20 | 2 | 0.100 | 0.095 | -0.027 | 0 |
| MA39_185 | 20 | 9 | 0.750 | 0.758 | 0.036 | 0 |
| MA39_199 | 19 | 2 | 0.211 | 0.188 | -0.091 | 0 |
| MA39_442 | 20 | 2 | 0.100 | 0.095 | -0.027 | 0 |
| MA40_045 | 20 | 9 | 0.800 | 0.809 | 0.037 | 0 |
| MA40_282 | 20 | 6 | 0.650 | 0.605 | -0.049 | 0 |
| MA41_076 | 20 | 3 | 0.500 | 0.454 | -0.077 | 0 |
| MA41_264 | 20 | 4 | 0.850 | 0.588 | -0.426* | 0 |
| MA41_373 | 20 | 7 | 0.600 | 0.578 | -0.013 | 0 |
| MA42_001 | 20 | 5 | 0.700 | 0.709 | 0.038 | 0 |
| MA42_028^1^ | 19* | 7 | 0.474 | 0.785 | 0.419** | 0.236* |
| MA42_063 | 20 | 4 | 0.650 | 0.611 | -0.038 | 0 |
| MA42_077 | 20 | 3 | 0.300 | 0.329 | 0.113 | 0.029 |
| MA42_083 | 20 | 9 | 0.750 | 0.790 | 0.076 | 0.020 |
| MA42_087^2^ | 20 | 5 | 0.400 | 0.553 | 0.300* | 0.074 |
| MA42_166 | 20 | 4 | 0.600 | 0.614 | 0.048 | 0 |
| MA42_197 | 20 | 11 | 0.850 | 0.829 | 0.000 | 0.020 |
| MA42_203 | 20 | 8 | 0.750 | 0.808 | 0.097 | 0.027 |
| MA42_231 | 20 | 3 | 0.400 | 0.505 | 0.232 | 0.094 |
| MA42_241 | 20 | 2 | 0.550 | 0.439 | -0.229 | 0 |
| MA42_247 | 20 | 11 | 0.850 | 0.866 | 0.044 | 0 |
| MA42_255^2^ | 20 | 7 | 0.550 | 0.740 | 0.281* | 0.067 |
| MA42_274 | 20 | 7 | 0.700 | 0.710 | 0.040 | 0 |
| MA42_296 | 20 | 6 | 0.600 | 0.719 | 0.190 | 0.051 |
| MA42_397 | 20 | 11 | 0.850 | 0.891 | 0.072 | 0.032 |
| MA42_413 | 20 | 3 | 0.400 | 0.366 | -0.067 | 0 |
| MA42_421 | 20 | 7 | 0.700 | 0.818 | 0.169 | 0.054 |
| MA42_471 | 20 | 5 | 0.550 | 0.648 | 0.176 | 0.067 |
| MA42_472 | 20 | 8 | 0.900 | 0.834 | -0.054 | 0 |
| MA42_481 | 20 | 8 | 0.750 | 0.825 | 0.116 | 0.023 |
| **Pop (30^1^)** | 19.967 | 5.833 | 0.597 | 0.613 | 0.052 |  |
| **SD (30^1^)** | 0.033 | 0.521 | 0.040 | 0.041 |  |  |
| **Pop (28^1,2^)** | 19.964 | 5.821 | 0.606 | 0.611 | 0.034 |  |
| **SD (28^1,2^)** | 0.036 | 0.556 | 0.042 | 0.043 |  |  |

**Supplementary Table S4c**: *Magnolia dodecapetala*: 21 polymorphic SSR markers.

| **Population** | MART | | | | | | GUA | | | | | |
| --- | --- | --- | --- | --- | --- | --- | --- | --- | --- | --- | --- | --- |
| **SSR marker** | **N** | **A** | **H_o_** | **H_e_** | **F_is_** | **A_0_** | **N** | **A** | **H_o_** | **H_e_** | **F_is_** | **A_0_** |
| MA39_023 | 20 | 11 | 0.850 | 0.831 | 0.003 | 0 | 20 | 2 | 0.250 | 0.219 | -0.118 | 0 |
| MA39_159 | 20 | 2 | 0.350 | 0.399 | 0.147 | 0.040 | 20 | 3 | 0.100 | 0.096 | -0.013 | 0 |
| MA39_182 | 20 | 2 | 0.350 | 0.399 | 0.147 | 0.040 | 20 | 2 | 0.300 | 0.375 | 0.224 | 0.064 |
| MA39_185 | 19 | 8 | 0.789 | 0.803 | 0.044 | 0.010 | 20 | 8 | 0.700 | 0.810 | 0.161 | 0.068 |
| MA39_199^1^ | 20 | 2 | 0.150 | 0.139 | -0.056 | 0 | 19* | 3 | 0.053 | 0.445 | 0.888** | 0.352* |
| MA39_259^2^ | 20 | 2 | 0.050 | 0.049 | 0 | 0 | 20 | 2 | 0.000 | 0.095 | 1.000* | 0.149* |
| MA39_287^2^ | 20 | 10 | 0.650 | 0.834 | 0.245* | 0.090* | 20 | 9 | 0.700 | 0.778 | 0.125 | 0.015 |
| MA39_442 | 20 | 2 | 0.050 | 0.049 | 0 | 0 | 20 | 1 | 0.000 | 0.000 | M | 0 |
| MA40_136 | 20 | 1 | 0.000 | 0.000 | M | 0 | 20 | 3 | 0.500 | 0.535 | 0.091 | 0.014 |
| MA40_282^2^ | 20 | 8 | 0.450 | 0.724 | 0.400** | 0.147* | 20 | 10 | 0.750 | 0.838 | 0.130* | 0.022 |
| MA42_072 | 20 | 2 | 0.250 | 0.219 | -0.118 | 0 | 19 | 3 | 0.263 | 0.342 | 0.256 | 0.088 |
| MA42_077^2^ | 20 | 13 | 0.500 | 0.825 | 0.415** | 0.182* | 20 | 13 | 0.950 | 0.895 | -0.036 | 0 |
| MA42_231 | 20 | 12 | 0.850 | 0.863 | 0.040 | 0 | 20 | 8 | 0.750 | 0.821 | 0.112 | 0.058 |
| MA42_255 | 20 | 6 | 0.900 | 0.710 | -0.244 | 0 | 20 | 10 | 0.850 | 0.860 | 0.037 | 0 |
| MA42_274 | 20 | 14 | 0.900 | 0.910 | 0.037 | 0 | 20 | 15 | 0.850 | 0.878 | 0.057* | 0.042 |
| MA42_333 | 20 | 7 | 0.650 | 0.766 | 0.177 | 0.032 | 20 | 18 | 0.950 | 0.909 | -0.020 | 0 |
| MA42_372^1^ | 19 | 15 | 0.526 | 0.909 | 0.443** | 0.245* | 20 | 8 | 0.800 | 0.784 | 0.005 | 0.005 |
| MA42_397^1^ | 19 | 3 | 0.211 | 0.652 | 0.692** | 0.326* | 20 | 3 | 0.300 | 0.516 | 0.440 | 0.139* |
| MA42_421 | 20 | 1 | 0.000 | 0.000 | M | 0 | 20 | 2 | 0.100 | 0.095 | -0.027 | 0 |
| MA42_471 | 20 | 18 | 0.900 | 0.910 | 0.037 | 0 | 20 | 13 | 0.800 | 0.865 | 0.101 | 0.037 |
| MA42_495 | 20 | 2 | 0.100 | 0.095 | -0.027 | 0 | 20 | 16 | 0.850 | 0.881 | 0.061 | 0.003 |
| **Pop (21)** | 19.857 | 6.714 | 0.451 | 0.528 | 0.170* |  | 19.905 | 7.238 | 0.515 | 0.573 | 0.127* |  |
| **SD (21)** | 0.078 | 1.179 | 0.072 | 0.078 |  |  | 0.066 | 1.173 | 0.075 | 0.071 |  |  |
| **Pop (18^1^)** | 19.944 | 6.722 | 0.477 | 0.521 | 0.110* |  | 19.944 | 7.667 | 0.537 | 0.572 | 0.087 |  |
| **SD (18^1^)** | 0.056 | 1.252 | 0.081 | 0.085 |  |  | 0.056 | 1.326 | 0.081 | 0.082 |  |  |
| **Pop (14^1,2^)** | 19.929 | 6.286 | 0.496 | 0.497 | 0.028 |  | 19.929 | 7.429 | 0.519 | 0.549 | 0.081 |  |
| **SD (14^1,2^)** | 0.071 | 1.488 | 0.099 | 0.098 |  |  | 0.071 | 1.606 | 0.090 | 0.093 |  |  |

**Supplementary Table S4d**: *Magnolia domingensis:* 21 polymorphic microsatellite markers. Null alleles: MA39_199. Allelic associations: 43/210 pairwise tests with a p-value lower than 0.05, of which two pairs: MA39_199 × MA42_421 (indicated with A1) and MA42_231 × MA42_472 (indicated with A2) remained significant after sequential Bonferroni corrections. There were 210 pairwise tests, 10.5 [6, 16] expected to test false positive when p = 0.05. When considering the two populations separately, 15/210 and 41/210 pairwise tests were significant for BAR and ROD, respectively.

| **Population** | BAR | | | | | | ROD | | | | | |
| --- | --- | --- | --- | --- | --- | --- | --- | --- | --- | --- | --- | --- |
| **SSR marker** | **N** | **A** | **H_o_** | **H_e_** | **F_is_** | **A_0_** | **N** | **A** | **H_o_** | **H_e_** | **F_is_** | **A_0_** |
| MA39_023 | 20 | 4 | 0.600 | 0.621 | 0.06 | 0.004 | 20 | 4 | 0.800 | 0.666 | -0.176* | 0 |
| MA39_165 | 20 | 1 | 0.000 | 0.000 | M | 0 | 20 | 2 | 0.250 | 0.439 | 0.451 | 0.138 |
| MA39_185 | 20 | 5 | 0.850 | 0.759 | -0.095 | 0 | 20 | 4 | 0.750 | 0.528 | -0.4 | 0 |
| MA39_199^1(A1)^ | 20 | 4 | 0.350 | 0.539 | 0.373* | 0.126* | 20 | 3 | 0.350 | 0.301 | -0.137 | 0 |
| MA40_282 | 20 | 6 | 1.000 | 0.794 | -0.236 | 0 | 20 | 6 | 0.850 | 0.729 | -0.141 | 0 |
| MA41_373 | 20 | 6 | 0.700 | 0.679 | -0.006 | 0.014 | 20 | 5 | 0.800 | 0.746 | -0.046 | 0 |
| MA42_001 | 20 | 5 | 0.750 | 0.694 | -0.056 | 0 | 20 | 2 | 0.250 | 0.219 | -0.118 | 0 |
| MA42_059 | 20 | 2 | 0.550 | 0.399 | -0.357 | 0 | 20 | 1 | 0.000 | 0.000 | M | 0 |
| MA42_077 | 20 | 3 | 0.500 | 0.486 | -0.003 | 0 | 20 | 2 | 0.350 | 0.489 | 0.307 | 0.094 |
| MA42_126 | 20 | 2 | 0.500 | 0.455 | -0.073 | 0 | 20 | 2 | 0.600 | 0.480 | -0.226 | 0 |
| MA42_166 | 20 | 3 | 0.400 | 0.486 | 0.202 | 0.049 | 20 | 3 | 0.150 | 0.141 | -0.036 | 0 |
| MA42_203 | 20 | 5 | 0.800 | 0.609 | -0.291 | 0 | 20 | 3 | 0.550 | 0.514 | -0.045 | 0 |
| MA42_231^A2^ | 20 | 5 | 0.750 | 0.754 | 0.031 | 0 | 20 | 5 | 0.650 | 0.715 | 0.116 | 0.018 |
| MA42_255 | 20 | 4 | 0.850 | 0.675 | -0.235 | 0 | 20 | 4 | 0.550 | 0.581 | 0.079 | 0.017 |
| MA42_293 | 20 | 2 | 0.600 | 0.455 | -0.295 | 0 | 20 | 2 | 0.050 | 0.049 | 0 | 0 |
| MA42_334 | 20 | 2 | 0.400 | 0.480 | 0.191 | 0 | 20 | 2 | 0.250 | 0.219 | -0.118 | 0 |
| MA42_397 | 20 | 11 | 0.850 | 0.835 | 0.008 | 0.055 | 20 | 5 | 0.800 | 0.668 | -0.174* | 0 |
| MA42_421^A1^ | 20 | 4 | 0.450 | 0.446 | 0.017 | 0 | 20 | 2 | 0.450 | 0.499 | 0.123 | 0 |
| MA42_472^1(A2)^ | 20 | 10 | 0.750 | 0.828 | 0.119 | 0.026 | 20 | 8 | 0.950 | 0.793 | -0.174 | 0 |
| MA42_481 | 20 | 8 | 0.850 | 0.768 | -0.082 | 0 | 20 | 6 | 0.750 | 0.826 | 0.118 | 0.035 |
| MA42_495 | 19 | 3 | 0.474 | 0.492 | 0.064 | 0.016 | 20 | 4 | 0.700 | 0.648 | -0.056 | 0 |
| **Pop (19^1^)** | 19.947 | 4.263 | 0.625 | 0.573 | -0.065 |  | 20 | 3.368 | 0.503 | 0.482 | -0.018 |  |
| **SD (19^1^)** | 0.053 | 0.551 | 0.054 | 0.045 |  |  | 0 | 0.352 | 0.064 | 0.056 |  |  |

**Supplementary Table S4e**: *Magnolia ekmanii*: 30 polymorphic SSR markers.

| **Population** | GRA | | | | | | MAN | | | | | |
| --- | --- | --- | --- | --- | --- | --- | --- | --- | --- | --- | --- | --- |
| **SSR marker** | **N** | **A** | **H_o_** | **H_e_** | **F_is_** | **A_0_** | **N** | **A** | **H_o_** | **H_e_** | **F_is_** | **A_0_** |
| MA39_023^1^ | 20 | 4 | 0.200 | 0.596 | 0.679** | 0.240* | 19 | 3 | 0.474 | 0.566 | 0.190 | 0.125 |
| MA39_185 | 20 | 2 | 0.150 | 0.219 | 0.337 | 0.085 | 20 | 5 | 0.700 | 0.731 | 0.068 | 0 |
| MA39_236 | 20 | 7 | 0.700 | 0.693 | 0.015* | 0 | 20 | 8 | 0.750 | 0.686 | -0.067 | 0 |
| MA39_259 | 20 | 2 | 0.200 | 0.180 | -0.086 | 0 | 20 | 2 | 0.500 | 0.495 | 0.016 | 0 |
| MA39_263 | 20 | 1 | 0.000 | 0.000 | M | 0 | 20 | 2 | 0.500 | 0.420 | -0.166 | 0 |
| MA40_072 | 20 | 2 | 0.050 | 0.049 | 0 | 0 | 20 | 2 | 0.550 | 0.499 | -0.077 | 0 |
| MA40_282 | 20 | 4 | 0.750 | 0.714 | -0.025 | 0 | 20 | 3 | 0.450 | 0.454 | 0.034 | 0 |
| MA41_076 | 20 | 7 | 0.650 | 0.695 | 0.090 | 0 | 20 | 3 | 0.250 | 0.366 | 0.340 | 0.103 |
| MA41_215 | 20 | 1 | 0.000 | 0.000 | M | 0 | 20 | 2 | 0.400 | 0.420 | 0.073 | 0.016 |
| MA41_264 | 20 | 2 | 0.300 | 0.320 | 0.088 | 0.020 | 20 | 2 | 0.100 | 0.180 | 0.465 | 0.106 |
| MA41_373 | 20 | 5 | 0.550 | 0.546 | 0.019 | 0 | 20 | 1 | 0.000 | 0.000 | M | 0 |
| MA42_001 | 20 | 4 | 0.700 | 0.580 | -0.182 | 0 | 20 | 3 | 0.550 | 0.540 | 0.007 | 0 |
| MA42_028 | 20 | 1 | 0.000 | 0.000 | M | 0 | 20 | 2 | 0.350 | 0.349 | 0.022 | 0 |
| MA42_077 | 20 | 3 | 0.550 | 0.526 | -0.020 | 0 | 20 | 2 | 0.300 | 0.255 | -0.152 | 0 |
| MA42_083 | 20 | 3 | 0.300 | 0.261 | -0.123 | 0 | 20 | 2 | 0.250 | 0.219 | -0.118 | 0 |
| MA42_087^1^ | 20 | 5 | 0.350 | 0.585 | 0.423* | 0.119* | 20 | 4 | 0.600 | 0.616 | 0.052 | 0 |
| MA42_102 | 20 | 5 | 0.750 | 0.700 | -0.046 | 0 | 20 | 6 | 0.850 | 0.800 | -0.037 | 0 |
| MA42_202 | 20 | 12 | 0.950 | 0.893 | -0.039 | 0 | 20 | 9 | 1.000 | 0.774 | -0.269 | 0 |
| MA42_203 | 20 | 4 | 0.650 | 0.656 | 0.035 | 0 | 20 | 3 | 0.550 | 0.526 | -0.020 | 0 |
| MA42_231 | 20 | 3 | 0.100 | 0.096 | -0.013 | 0 | 20 | 1 | 0.000 | 0.000 | M | 0 |
| MA42_247 | 20 | 8 | 0.950 | 0.798 | -0.166 | 0 | 20 | 5 | 0.400 | 0.433 | 0.101 | 0.003 |
| MA42_253 | 20 | 6 | 0.600 | 0.614 | 0.048 | 0 | 20 | 6 | 0.700 | 0.756 | 0.100 | 0.022 |
| MA42_255 | 20 | 3 | 0.100 | 0.096 | -0.013 | 0 | 20 | 3 | 0.500 | 0.576 | 0.157 | 0.059 |
| MA42_397 | 20 | 9 | 0.900 | 0.826 | -0.064 | 0 | 19 | 10 | 1.000 | 0.778 | -0.260 | 0 |
| MA42_413 | 20 | 4 | 0.500 | 0.516 | 0.057 | 0 | 20 | 2 | 0.050 | 0.049 | 0 | 0 |
| MA42_421 | 20 | 6 | 0.750 | 0.695 | -0.054 | 0 | 20 | 3 | 0.600 | 0.629 | 0.071 | 0.008 |
| MA42_472 | 20 | 4 | 0.300 | 0.270 | -0.086 | 0 | 20 | 4 | 0.300 | 0.269 | -0.091 | 0 |
| MA42_481 | 20 | 4 | 0.550 | 0.569 | 0.059 | 0 | 20 | 2 | 0.100 | 0.095 | -0.027 | 0 |
| MA42_491 | 20 | 6 | 0.650 | 0.665 | 0.048 | 0 | 20 | 5 | 0.650 | 0.681 | 0.071 | 0 |
| MA42_495 | 20 | 9 | 0.850 | 0.820 | -0.011 | 0 | 19 | 8 | 0.947 | 0.838 | -0.104 | 0 |
| **Pop (28^1^)** | 20 | 4.536 | 0.482 | 0.464 | -0.013 |  | 19.929 | 3.786 | 0.475 | 0.458 | -0.012 |  |
| **SD (28^1^)** | 0 | 0.516 | 0.059 | 0.055 |  |  | 0.050 | 0.470 | 0.055 | 0.048 |  |  |

**Supplementary Table S4f**: *Magnolia hamorii*: 24 polymorphic SSR markers.

| **Population** | COR | | | | | | CAC | | | | | |
| --- | --- | --- | --- | --- | --- | --- | --- | --- | --- | --- | --- | --- |
| **SSR marker** | **N** | **A** | **H_o_** | **H_e_** | **F_is_** | **A_0_** | **N** | **A** | **H_o_** | **H_e_** | **F_is_** | **A_0_** |
| MA39_023 | 20 | 2 | 0.300 | 0.480 | 0.397 | 0.124 | 20 | 2 | 0.450 | 0.349 | -0.267 | 0 |
| MA39_185 | 20 | 6 | 0.650 | 0.753 | 0.161 | 0.051 | 20 | 6 | 0.550 | 0.733 | 0.273 | 0.091 |
| MA39_199 | 20 | 2 | 0.250 | 0.219 | -0.118 | 0 | 20 | 3 | 0.400 | 0.516 | 0.249 | 0.072 |
| MA39_236 | 20 | 5 | 0.750 | 0.706 | -0.036 | 0 | 20 | 7 | 0.850 | 0.771 | -0.077* | 0 |
| MA40_045 | 20 | 12 | 0.900 | 0.878 | 0 | 0.004 | 20 | 11 | 0.950 | 0.883 | -0.051 | 0 |
| MA40_223^1^ | 20 | 2 | 0.250 | 0.489 | 0.508* | 0.162* | 20 | 2 | 0.500 | 0.375 | -0.31 | 0 |
| MA40_282 | 20 | 13 | 0.800 | 0.898 | 0.134 | 0.051 | 20 | 14 | 0.900 | 0.851 | -0.032 | 0 |
| MA41_215 | 20 | 5 | 0.900 | 0.769 | -0.146 | 0 | 20 | 6 | 0.800 | 0.755 | -0.034 | 0 |
| MA41_373 | 20 | 8 | 0.850 | 0.824 | -0.006 | 0 | 20 | 8 | 0.800 | 0.830 | 0.062 | 0 |
| MA42_001 | 20 | 3 | 0.450 | 0.511 | 0.145 | 0.035 | 20 | 3 | 0.500 | 0.564 | 0.138 | 0.077 |
| MA42_077 | 20 | 2 | 0.550 | 0.489 | -0.1 | 0 | 20 | 2 | 0.450 | 0.469 | 0.066 | 0.013 |
| MA42_083 | 20 | 5 | 0.750 | 0.691 | -0.059 | 0 | 20 | 5 | 0.700 | 0.689 | 0.009 | 0 |
| MA42_102 | 20 | 9 | 0.900 | 0.861 | -0.019 | 0 | 20 | 8 | 0.800 | 0.813 | 0.041 | 0 |
| MA42_126 | 20 | 4 | 0.550 | 0.579 | 0.075 | 0 | 20 | 2 | 0.600 | 0.480 | -0.226 | 0 |
| MA42_203 | 20 | 6 | 0.700 | 0.786 | 0.135 | 0.041 | 20 | 6 | 0.700 | 0.705 | 0.033 | 0.003 |
| MA42_231 | 20 | 6 | 0.850 | 0.775 | -0.071 | 0 | 20 | 7 | 0.850 | 0.776 | -0.07 | 0 |
| MA42_241 | 20 | 2 | 0.750 | 0.499 | -0.484 | 0 | 20 | 2 | 0.300 | 0.375 | 0.224 | 0.064 |
| MA42_255 | 20 | 7 | 0.850 | 0.823 | -0.008 | 0 | 20 | 9 | 0.800 | 0.835 | 0.067* | 0.031 |
| MA42_296 | 20 | 8 | 0.850 | 0.760 | -0.093 | 0 | 20 | 5 | 0.600 | 0.750 | 0.224 | 0.089 |
| MA42_397 | 20 | 10 | 0.800 | 0.825 | 0.056 | 0 | 20 | 9 | 0.800 | 0.844 | 0.077 | 0.001 |
| MA42_413^1^ | 20 | 8 | 0.600 | 0.798 | 0.272* | 0.114* | 20 | 6 | 0.500 | 0.711 | 0.320 | 0.116 |
| MA42_471 | 20 | 7 | 0.850 | 0.804 | -0.032 | 0 | 20 | 6 | 0.900 | 0.803 | -0.096 | 0 |
| MA42_472 | 20 | 16 | 0.950 | 0.905 | -0.024 | 0 | 20 | 15 | 0.950 | 0.896 | -0.034 | 0 |
| MA42_481 | 20 | 9 | 0.700 | 0.833 | 0.184 | 0.089 | 20 | 9 | 0.900 | 0.796 | -0.105 | 0 |
| **Pop (22^1^)** | 20 | 6.682 | 0.723 | 0.712 | 0.011 |  | 20 | 6.591 | 0.707 | 0.704 | 0.021 |  |
| **SD (22^1^)** | 0 | 0.804 | 0.041 | 0.037 |  |  | 0 | 0.783 | 0.042 | 0.036 |  |  |

**Supplementary Table S4g**: *Magnolia lacandonica*: 23 polymorphic SSR markers. Allelic associations: there were 36/231 pairwise tests with a p-value lower than 0.05. Two pairs: MA39_185 × MA39_442 (A1) and MA41_373 × MA42_028 (A2) remained significant after sequential Bonferroni corrections. Of the 231 pairwise tests, 12.65 [7, 19] were expected to test false positive when p = 0.05. When the populations were considered separately, the high amount of allelic association remains: 27/231 for LAC and 33/231 for YAJ.

| **Population** | LAC | | | | | | YAJ | | | | | |
| --- | --- | --- | --- | --- | --- | --- | --- | --- | --- | --- | --- | --- |
| **SSR marker** | **N** | **A** | **H_o_** | **H_e_** | **F_is_** | **A_0_** | **N** | **A** | **H_o_** | **H_e_** | **F_is_** | **A_0_** |
| MA39_023 | 20 | 1 | 0.000 | 0.000 | M | 0 | 20 | 2 | 0.300 | 0.320 | 0.088 | 0.020 |
| MA39_142 | 20 | 2 | 0.300 | 0.255 | -0.152 | 0 | 20 | 2 | 0.300 | 0.255 | -0.152 | 0 |
| MA39_159 | 20 | 7 | 0.900 | 0.828 | -0.062 | 0 | 20 | 4 | 0.600 | 0.509 | -0.154 | 0 |
| MA39_182^1^ | 19* | 5 | 0.474 | 0.715 | 0.361* | 0.227* | 20 | 4 | 0.150 | 0.404 | 0.644* | 0.192* |
| MA39_185^(A1)^ | 20 | 5 | 0.650 | 0.560 | -0.136 | 0 | 20 | 5 | 0.750 | 0.664 | -0.105* | 0 |
| MA39_236 | 20 | 7 | 0.800 | 0.825 | 0.056 | 0.030 | 20 | 7 | 1.000 | 0.768 | -0.279 | 0 |
| MA39_259 | 20 | 3 | 0.350 | 0.515 | 0.343 | 0.100 | 20 | 3 | 0.750 | 0.526 | -0.404 | 0 |
| MA39_287 | 20 | 4 | 0.800 | 0.678 | -0.156 | 0 | 20 | 7 | 0.900 | 0.766 | -0.15 | 0 |
| MA39_327 | 20 | 4 | 0.800 | 0.739 | -0.057 | 0 | 20 | 6 | 0.850 | 0.691 | -0.205 | 0 |
| MA39_342 | 20 | 7 | 0.900 | 0.814 | -0.081 | 0 | 20 | 9 | 0.850 | 0.799 | -0.039 | 0 |
| MA39_348 | 20 | 5 | 0.700 | 0.701 | 0.027 | 0.017 | 20 | 4 | 0.650 | 0.646 | 0.02 | 0.016 |
| MA39_442^1(A1)^ | 20 | 2 | 0.350 | 0.399 | 0.147 | 0.040 | 20 | 2 | 0.500 | 0.480 | -0.016 | 0 |
| MA40_072 | 20 | 3 | 0.250 | 0.335 | 0.278 | 0.059 | 20 | 6 | 0.950 | 0.770 | -0.209 | 0 |
| MA40_282 | 20 | 5 | 0.750 | 0.738 | 0.009 | 0 | 20 | 6 | 0.800 | 0.631 | -0.243 | 0 |
| MA41_076 | 20 | 8 | 0.800 | 0.749 | -0.043 | 0 | 20 | 6 | 0.650 | 0.754 | 0.163 | 0.076 |
| MA41_373^(A2)^ | 20 | 5 | 0.800 | 0.686 | -0.141 | 0 | 20 | 5 | 0.850 | 0.771 | -0.077 | 0 |
| MA42_028^1(A2)^ | 20 | 6 | 0.650 | 0.710 | 0.110* | 0 | 20 | 4 | 0.750 | 0.681 | -0.075 | 0 |
| MA42_231 | 20 | 5 | 0.800 | 0.725 | -0.078 | 0 | 20 | 4 | 0.250 | 0.269 | 0.095 | 0 |
| MA42_255 | 20 | 4 | 0.700 | 0.666 | -0.025 | 0.016 | 20 | 4 | 0.600 | 0.486 | -0.21 | 0 |
| MA42_274 | 20 | 4 | 0.650 | 0.621 | -0.021 | 0 | 20 | 5 | 0.850 | 0.703 | -0.185 | 0 |
| MA42_421 | 20 | 3 | 0.650 | 0.554 | -0.149 | 0 | 20 | 4 | 0.850 | 0.676 | -0.233 | 0 |
| MA42_471 | 20 | 6 | 0.750 | 0.749 | 0.024 | 0 | 20 | 4 | 0.550 | 0.446 | -0.208 | 0 |
| MA42_495 | 20 | 2 | 0.400 | 0.320 | -0.226 | 0 | 20 | 2 | 0.450 | 0.399 | -0.103 | 0 |
| **Pop (20^1^)** | 20 | 4.500 | 0.638 | 0.603 | -0.032 |  | 20 | 4.750 | 0.688 | 0.592 | -0.135 |  |
| **SD (20^1^)** | 0 | 0.420 | 0.055 | 0.049 |  |  | 0 | 0.410 | 0.050 | 0.040 |  |  |

**Supplementary Table S4h**: *Magnolia pallescens*: 20 polymorphic SSR markers.

| **Population** | SAL | | | | | | MON | | | | | |
| --- | --- | --- | --- | --- | --- | --- | --- | --- | --- | --- | --- | --- |
| **SSR marker** | **N** | **A** | **Ho** | **He** | **Fis** | **A_0_** | **N** | **A** | **Ho** | **He** | **Fis** | **A_0_** |
| MA39_023^1^ | 20 | 5 | 0.400 | 0.569 | 0.320* | 0.130* | 20 | 3 | 0.550 | 0.454 | -0.188 | 0 |
| MA39_185 | 20 | 6 | 0.700 | 0.813 | 0.164 | 0.065 | 20 | 5 | 0.650 | 0.641 | 0.012 | 0.031 |
| MA39_199 | 20 | 2 | 0.450 | 0.399 | -0.103 | 0 | 20 | 2 | 0.100 | 0.095 | -0.027 | 0 |
| MA39_259 | 20 | 2 | 0.100 | 0.095 | -0.027 | 0 | 20 | 2 | 0.400 | 0.375 | -0.041 | 0 |
| MA40_045^2^ (A1) | 20 | 6 | 0.850 | 0.766 | -0.084 | 0 | 20 | 5 | 0.500 | 0.644 | 0.180* | 0.088 |
| MA40_282 | 20 | 7 | 0.450 | 0.573 | 0.238 | 0.066 | 20 | 8 | 0.750 | 0.776 | 0.059 | 0 |
| MA41_373 | 20 | 4 | 0.500 | 0.569 | 0.146 | 0.064 | 20 | 3 | 0.500 | 0.591 | 0.179 | 0.057 |
| MA42_001 | 20 | 2 | 0.350 | 0.349 | 0.022 | 0 | 20 | 2 | 0.500 | 0.495 | 0.016 | 0 |
| MA42_059 | 20 | 2 | 0.250 | 0.219 | -0.118 | 0 | 20 | 1 | 0.000 | 0.000 | M | 0 |
| MA42_077 | 20 | 2 | 0.200 | 0.255 | 0.240 | 0.063 | 20 | 2 | 0.100 | 0.095 | -0.027 | 0 |
| MA42_083^2^ | 20 | 3 | 0.150 | 0.226 | 0.360* | 0.108 | 20 | 2 | 0.050 | 0.049 | 0 | 0 |
| MA42_203 | 20 | 6 | 0.800 | 0.728 | -0.074 | 0 | 20 | 5 | 0.650 | 0.630 | -0.006 | 0 |
| MA42_231 | 20 | 4 | 0.500 | 0.636 | 0.239 | 0.059 | 20 | 3 | 0.400 | 0.335 | -0.169 | 0 |
| MA42_241 | 20 | 1 | 0.000 | 0.000 | M | 0 | 20 | 2 | 0.250 | 0.289 | 0.159 | 0.042 |
| MA42_255 | 20 | 5 | 0.900 | 0.774 | -0.138 | 0 | 20 | 5 | 0.800 | 0.786 | 0.008 | 0 |
| MA42_293 | 20 | 3 | 0.300 | 0.265 | -0.107 | 0 | 20 | 2 | 0.450 | 0.499 | 0.123 | 0.033 |
| MA42_397 | 20 | 12 | 0.950 | 0.874 | -0.062 | 0 | 20 | 12 | 0.800 | 0.870 | 0.106 | 0.049 |
| MA42_421 | 20 | 7 | 0.900 | 0.814 | -0.081 | 0 | 20 | 7 | 0.650 | 0.719 | 0.121 | 0.042 |
| MA42_471 | 20 | 9 | 0.900 | 0.853 | -0.030 | 0 | 20 | 9 | 0.800 | 0.809 | 0.037 | 0 |
| MA42_472^1^ (A1) | 20 | 7 | 0.750 | 0.725 | -0.009 | 0 | 20 | 7 | 0.500 | 0.841 | 0.427** | 0.186* |
| **Pop (18^1^)** | 20 | 4.611 | 0.514 | 0.511 | 0.021 |  | 20 | 4.278 | 0.464 | 0.483 | 0.066 |  |
| **SD (18^1^)** | 0 | 0.687 | 0.074 | 0.068 |  |  | 0 | 0.713 | 0.064 | 0.067 |  |  |
| **Subset (16^1,2^)** | 20 | 4.625 | 0.516 | 0.513 | 0.018 |  | 20 | 4.375 | 0.488 | 0.500 | 0.045 |  |
| **SD (16^1,2^)** | 0 | 0.763 | 0.077 | 0.072 |  |  | 0 | 0.790 | 0.066 | 0.069 |  |  |

**Supplementary Table S4i**: *Magnolia portoricensis***:** 29 polymorphic SSR markers.

| **Population** | TOR | | | | | | MARI | | | | | |
| --- | --- | --- | --- | --- | --- | --- | --- | --- | --- | --- | --- | --- |
| **SSR marker** | **N** | **A** | **H_o_** | **H_e_** | **F_is_** | **A_0_** | **N** | **A** | **H_o_** | **H_e_** | **F_is_** | **A_0_** |
| MA39_023 | 20 | 8 | 0.700 | 0.776 | 0.124 | 0.034 | 20 | 7 | 0.700 | 0.779 | 0.126 | 0.069 |
| MA39_142 | 20 | 1 | 0.000 | 0.000 | M | 0 | 20 | 2 | 0.100 | 0.095 | -0.027 | 0 |
| MA39_185 | 20 | 14 | 0.700 | 0.868 | 0.218* | 0.086* | 20 | 7 | 0.700 | 0.755 | 0.098 | 0.045 |
| MA39_199 | 20 | 2 | 0.050 | 0.139 | 0.655 | 0.128 | 20 | 1 | 0.000 | 0.000 | M | 0 |
| MA39_236 | 20 | 6 | 0.750 | 0.791 | 0.078 | 0.012 | 20 | 3 | 0.400 | 0.340 | -0.152 | 0 |
| MA39_348 | 20 | 10 | 0.750 | 0.826 | 0.118 | 0.017 | 20 | 9 | 0.700 | 0.833 | 0.184 | 0.065 |
| MA40_045 | 20 | 8 | 0.600 | 0.766 | 0.241 | 0.080 | 20 | 8 | 0.800 | 0.786 | 0.008 | 0.013 |
| MA40_282 | 20 | 8 | 0.550 | 0.780 | 0.318* | 0.152* | 20 | 8 | 0.950 | 0.826 | -0.125 | 0 |
| MA41_076 | 20 | 3 | 0.100 | 0.096 | -0.013 | 0 | 20 | 2 | 0.050 | 0.049 | 0.000 | 0 |
| MA41_215 | 20 | 4 | 0.200 | 0.186 | -0.048 | 0 | 20 | 2 | 0.250 | 0.219 | -0.118 | 0 |
| MA41_373 | 20 | 7 | 0.700 | 0.765 | 0.110 | 0 | 20 | 9 | 0.800 | 0.838 | 0.070 | 0.017 |
| MA42_001 | 20 | 4 | 0.150 | 0.306 | 0.529* | 0.159* | 20 | 3 | 0.700 | 0.524 | -0.314 | 0 |
| MA42_063 | 20 | 8 | 0.750 | 0.801 | 0.089 | 0.009 | 19 | 10 | 0.684 | 0.828 | 0.200* | 0.082 |
| MA42_077 | 20 | 2 | 0.050 | 0.049 | 0.000 | 0 | 20 | 1 | 0.000 | 0.000 | M | 0 |
| MA42_087 | 20 | 6 | 0.600 | 0.701 | 0.169 | 0.061 | 20 | 7 | 0.750 | 0.795 | 0.082 | 0.041 |
| MA42_102 | 20 | 10 | 0.800 | 0.830 | 0.062 | 0.017 | 20 | 5 | 0.800 | 0.746 | -0.046 | 0 |
| MA42_126 | 20 | 3 | 0.200 | 0.445 | 0.568* | 0.181* | 20 | 4 | 0.550 | 0.638 | 0.162 | 0.036 |
| MA42_147 | 20 | 3 | 0.600 | 0.476 | -0.236 | 0 | 20 | 3 | 0.350 | 0.366 | 0.070 | 0.016 |
| MA42_185 | 20 | 9 | 0.750 | 0.838 | 0.130 | 0.039 | 20 | 6 | 0.800 | 0.745 | -0.048 | 0 |
| MA42_203 | 20 | 4 | 0.400 | 0.516 | 0.249 | 0.072 | 20 | 3 | 0.350 | 0.301 | -0.137 | 0 |
| MA42_231^2^ | 20 | 5 | 0.450 | 0.795 | 0.455** | 0.195* | 20 | 5 | 0.900 | 0.753 | -0.171 | 0 |
| MA42_255 | 20 | 4 | 0.650 | 0.681 | 0.071 | 0.037 | 20 | 7 | 0.650 | 0.735 | 0.141 | 0.054 |
| MA42_397 | 20 | 12 | 0.900 | 0.875 | -0.003 | 0 | 20 | 12 | 0.850 | 0.876 | 0.056 | 0 |
| MA42_413 | 20 | 4 | 0.350 | 0.584 | 0.422* | 0.137* | 20 | 2 | 0.100 | 0.095 | -0.027 | 0 |
| MA42_421 | 20 | 4 | 0.550 | 0.618 | 0.135 | 0.017 | 20 | 5 | 0.550 | 0.616 | 0.133 | 0.026 |
| MA42_471 | 20 | 9 | 0.900 | 0.824 | -0.067 | 0 | 20 | 9 | 0.800 | 0.800 | 0.026 | 0 |
| MA42_472 | 20 | 8 | 0.700 | 0.789 | 0.138 | 0.045 | 20 | 4 | 0.550 | 0.684 | 0.220 | 0.073 |
| MA42_481^1^ | 20 | 9 | 0.450 | 0.559 | 0.219* | 0.064 | 19* | 6 | 0.684 | 0.536 | -0.251 | 0 |
| MA42_495 | 20 | 10 | 0.800 | 0.863 | 0.098 | 0.037 | 20 | 6 | 1.000 | 0.761 | -0.290 | 0 |
| **Pop (28^1^)** | 20 | 6.286 | 0.525 | 0.607 | 0.160* |  | 19.964 | 5.357 | 0.566 | 0.564 | 0.022 |  |
| **SD (28^1^)** | 0 | 0.623 | 0.053 | 0.053 |  |  | 0.036 | 0.564 | 0.057 | 0.057 |  |  |

**Supplementary Table S4j**: *Magnolia splendens*: 25 polymorphic SSR markers.

| **Population** | YUN | | | | | |
| --- | --- | --- | --- | --- | --- | --- |
| **SSR marker** | **N_a_** | **A** | **H_o_** | **H_e_** | **F_is_** | **A_0_** |
| MA39_023^1^ | 20 | 13 | 0.600 | 0.790 | 0.265** | 0.127* |
| MA39_185 | 20 | 9 | 0.650 | 0.696 | 0.092 | 0 |
| MA39_348 | 19 | 11 | 0.850 | 0.880 | 0.060 | 0.009 |
| MA40_136 | 20 | 2 | 0.200 | 0.180 | -0.086 | 0 |
| MA40_175 | 20 | 2 | 0.250 | 0.399 | 0.395 | 0.117 |
| MA40_223 | 20 | 2 | 0.100 | 0.095 | -0.027 | 0 |
| MA40_282 | 20 | 8 | 0.850 | 0.825 | -0.005 | 0.020 |
| MA41_076 | 20 | 4 | 0.550 | 0.690 | 0.227 | 0.076 |
| MA41_373 | 20 | 7 | 0.800 | 0.795 | 0.019 | 0 |
| MA42_001 | 20 | 7 | 0.750 | 0.779 | 0.063 | 0 |
| MA42_063 | 20 | 6 | 0.800 | 0.766 | -0.018 | 0 |
| MA42_077 | 20 | 3 | 0.250 | 0.359 | 0.326 | 0.095 |
| MA42_102 | 20 | 8 | 0.800 | 0.830 | 0.062 | 0 |
| MA42_126 | 20 | 3 | 0.350 | 0.386 | 0.119 | 0.008 |
| MA42_147 | 19 | 6 | 0.737 | 0.734 | 0.023 | 0 |
| MA42_203 | 20 | 3 | 0.350 | 0.486 | 0.304 | 0.092 |
| MA42_231 | 20 | 6 | 0.750 | 0.710 | -0.031 | 0 |
| MA42_241 | 20 | 3 | 0.450 | 0.421 | -0.043 | 0 |
| MA42_255 | 20 | 7 | 0.850 | 0.783 | -0.061 | 0.035 |
| MA42_397 | 20 | 10 | 0.650 | 0.808 | 0.220 | 0.073 |
| MA42_413 | 20 | 4 | 0.650 | 0.631 | -0.004 | 0 |
| MA42_421 | 20 | 2 | 0.400 | 0.375 | -0.041 | 0 |
| MA42_471 | 20 | 4 | 0.700 | 0.528 | -0.304* | 0 |
| MA42_472 | 20 | 7 | 0.600 | 0.695 | 0.162 | 0.017 |
| MA42_481^1^ | 20 | 9 | 0.650 | 0.849 | 0.258 | 0.101* |
| **Pop (23^1^)** | 19.957 | 5.391 | 0.580 | 0.602 | 0.063 |  |
| **SD (23^1^)** | 0.043 | 0.572 | 0.049 | 0.046 |  |  |
