# Supplementary material for: Genetic patterns in Neotropical Magnolias (Magnoliaceae) using de novo developed microsatellite markers
Source: Heredity (Edinb). 2018 Oct 27;122(4):485–500. doi: 10.1038/s41437-018-0151-5 (PMC6460770; doi:10.1038/s41437-018-0151-5)
Supplement: Supplementary file 5 — Supplementary Figure S5 [file 41437_2018_151_MOESM5_ESM.pdf]

Supplementary Figure S5 STRUCTURE ΔK (Evanno et al., 2005) and mean likelihood plots. **A** dataset 1 which comprises 340 individuals representing 17 populations, genotyped for all 63 microsatellite markers where possible, including the assumed monomorphic data (See Supplementary Table S2: categories A, B and C). **B** dataset 2 which comprises 340 individuals representing 17 populations, genotyped for all 63 microsatellite markers where possible, excluding the assumed monomorphic data (See Supplementary Table S2: categories A and B). **C** dataset 3 which comprises 260 individuals representing 13 populations of the 8 taxa of the section *Talauma* subsection *Splendentes* (See Table 1: Class. = TAS), genotyped for 10 microsatellite markers (See Supplementary Table S2: marker names indicated with an asterisk). **D** DR-dataset comprising the 120 individuals representing 6 populations and 3 species of the Dominican Republic for all the markers of which data was generated (See Supplementary Table S2: categories A, B and C in the columns DOM, HAM and PAL); **D1**: analysis run with the independent allele model; **D2**: analysis run with the correlated allele model. **E** PR-dataset comprising 60 individuals representing three populations and two species of Puerto Rico for all the markers of which data was generated (See Supplementary Table S2: categories A, B and C in the columns POR and SPL); **E1**: analysis run with the independent allele model; **E2**: analysis run with the correlated allele model. **F** *Magnolia cubensis*. **G** *M. dodecapetala*. **H** *M. domingensis*. **I** *M. ekmanii*. **J** *M. hamorii*. **K** *M. lacandonica*. **L** *M. pallescens*. **M** *M. portoricensis*. **N** *M. splendens*. **O1** *M. cubensis* subsp. *acunae*. **O2** *M. cubensis* subsp. *cubensis*. **P1** *M. dodecapetala*: GUA population. **P2** *M. dodecapetala*: MART population. **Q1** *M. domingensis*: BAR population. **Q2** *M. domingensis*: ROD population. **R1** *M. ekmanii*: GRA population. **R2** *M. ekmanii*: MAN population. **S1** *M. hamorii*: CAC population. **S2** *M. hamorii*: COR population. **T1** *M. lacandonica*: LAC population. **T2** *M. lacandonica*: YAJ population. **U1** *M. pallescens*: MON population. **U2** *M. pallescens*: SAL population. **V1** *M. portoricensis*: MARI population. **V2** *M. portoricensis*: MARI population.

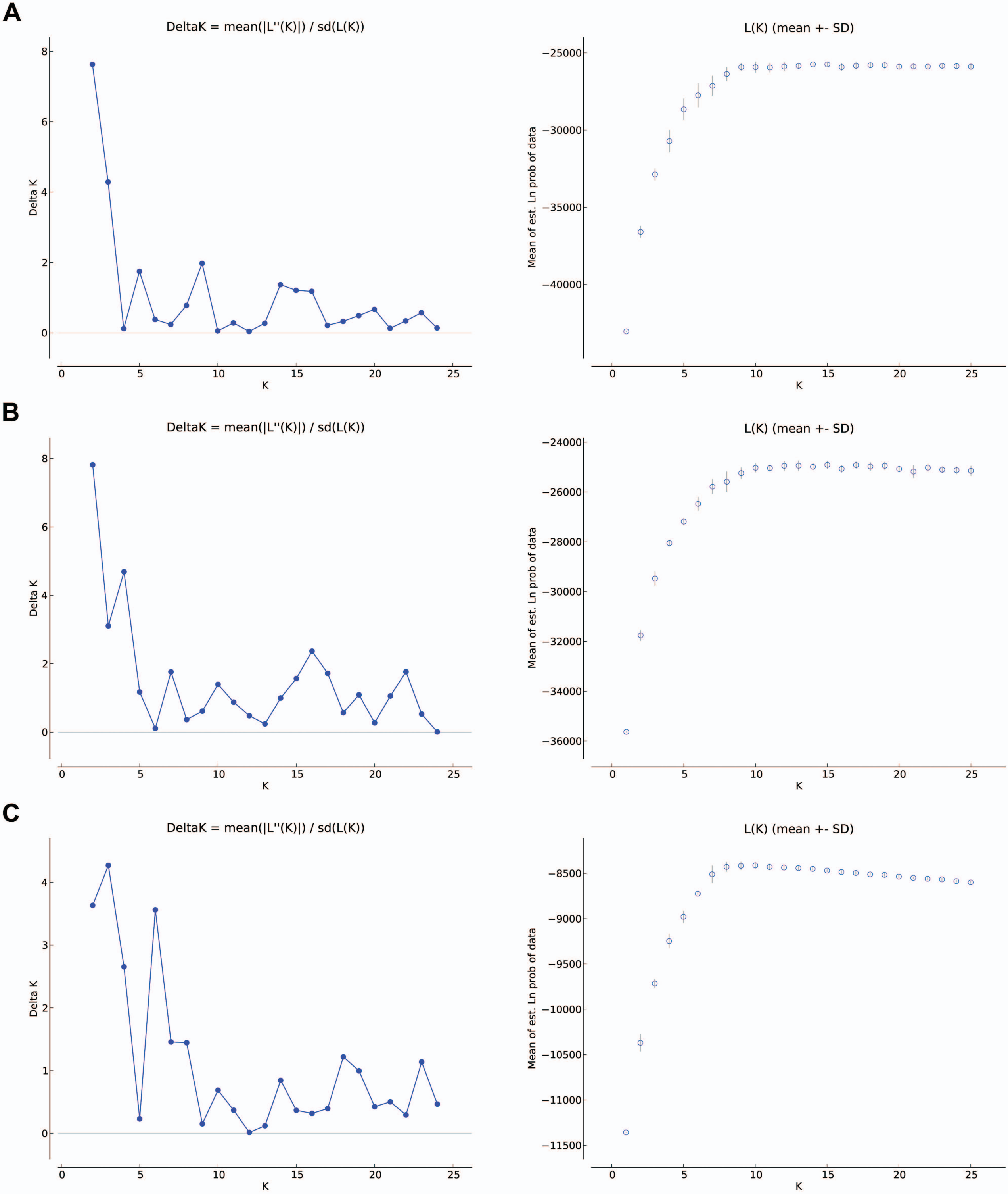
A
DeltaK = mean(|L''(K)|) / sd(L(K))
Delta K
K
L(K) (mean +- SD)
Mean of est. Ln prob of data
K
B
DeltaK = mean(|L''(K)|) / sd(L(K))
Delta K
K
L(K) (mean +- SD)
Mean of est. Ln prob of data
K
C
DeltaK = mean(|L''(K)|) / sd(L(K))
Delta K
K
L(K) (mean +- SD)
Mean of est. Ln prob of data
K

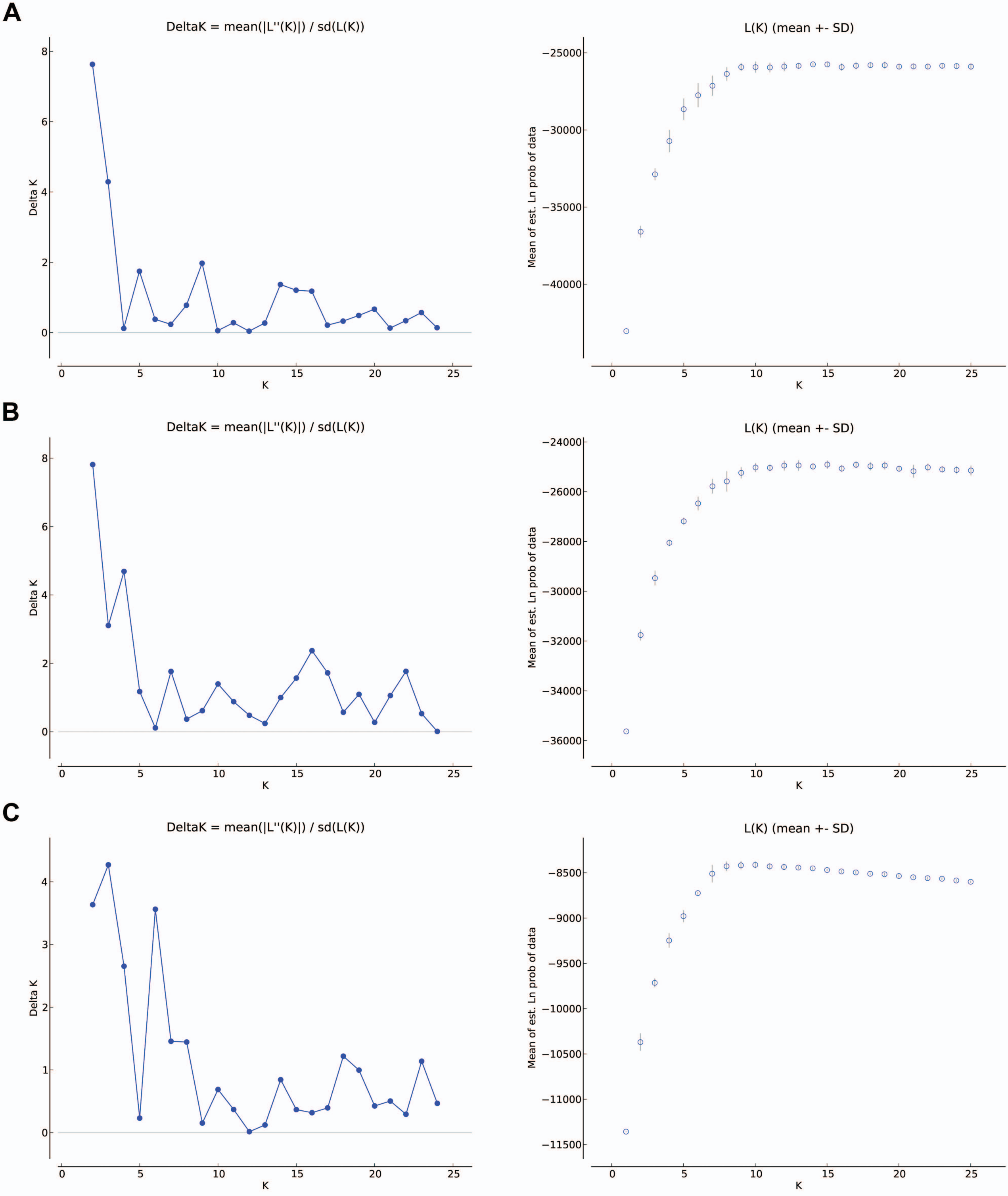

A
DeltaK = mean(|L''(K)|) / sd(L(K))
Delta K
K
L(K) (mean +- SD)
Mean of est. Ln prob of data
K
B
DeltaK = mean(|L''(K)|) / sd(L(K))
Delta K
K
L(K) (mean +- SD)
Mean of est. Ln prob of data
K
C
DeltaK = mean(|L''(K)|) / sd(L(K))
Delta K
K
L(K) (mean +- SD)
Mean of est. Ln prob of data
K

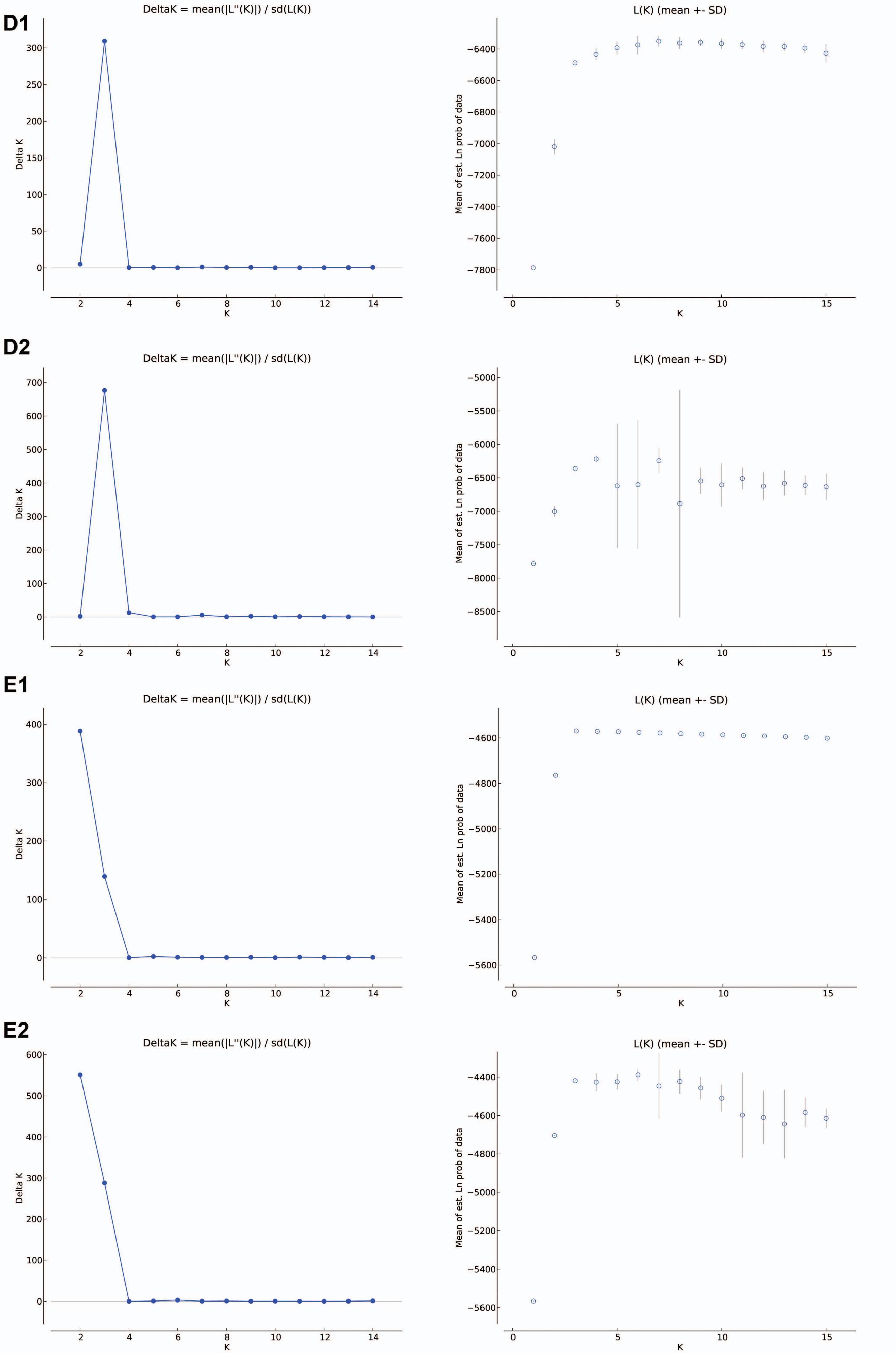
D1
DeltaK = mean(|L''(K)|) / sd(L(K))
Delta K
K
L(K) (mean +- SD)
Mean of est. Ln prob of data
K
D2
DeltaK = mean(|L''(K)|) / sd(L(K))
Delta K
K
L(K) (mean +- SD)
Mean of est. Ln prob of data
K
E1
DeltaK = mean(|L''(K)|) / sd(L(K))
Delta K
K
L(K) (mean +- SD)
Mean of est. Ln prob of data
K
E2
DeltaK = mean(|L''(K)|) / sd(L(K))
Delta K
K
L(K) (mean +- SD)
Mean of est. Ln prob of data
K

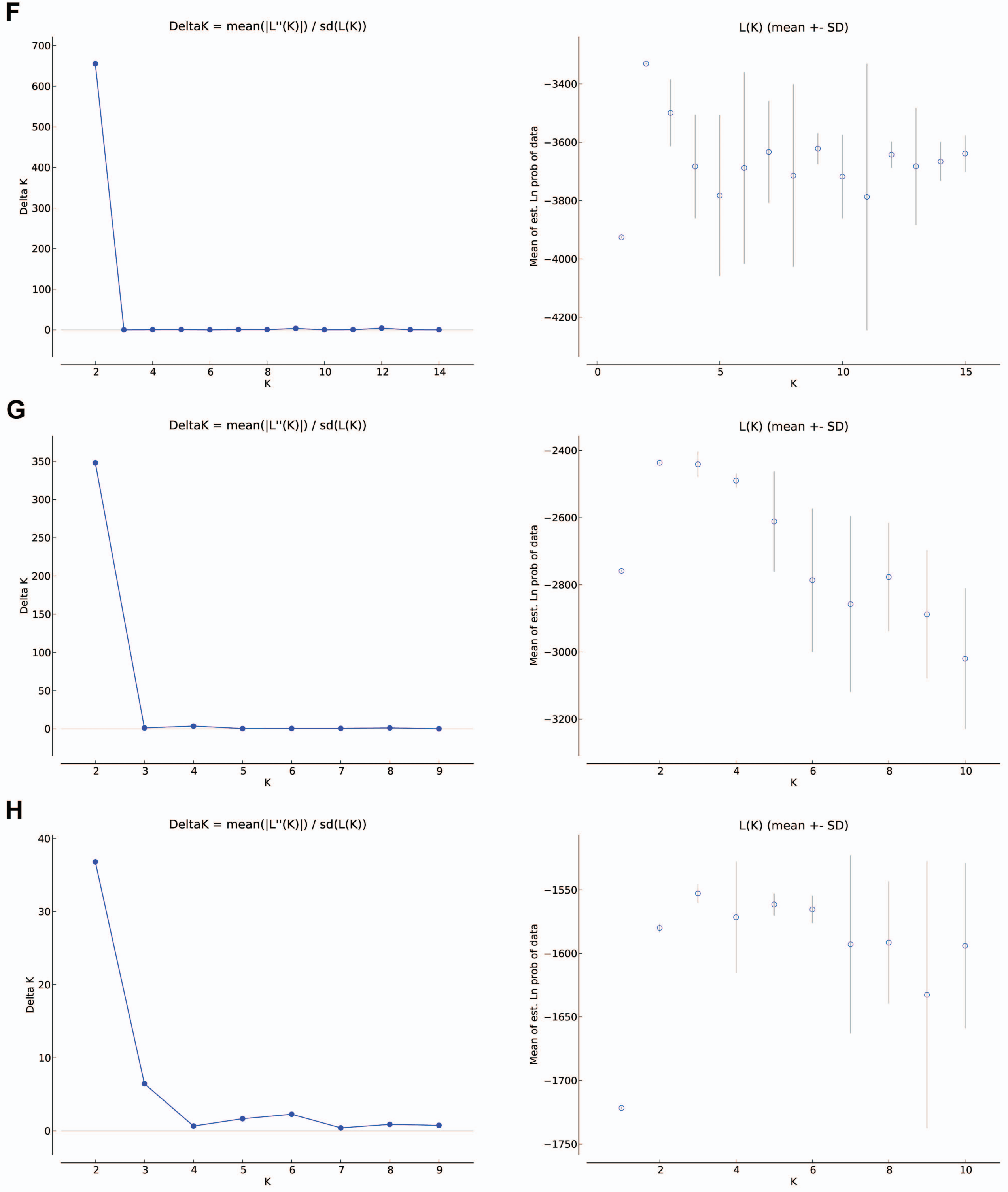
F
DeltaK = mean(|L''(K)|) / sd(L(K))
Delta K
K
L(K) (mean +- SD)
Mean of est. Ln prob of data
K
G
DeltaK = mean(|L''(K)|) / sd(L(K))
Delta K
K
L(K) (mean +- SD)
Mean of est. Ln prob of data
K
H
DeltaK = mean(|L''(K)|) / sd(L(K))
Delta K
K
L(K) (mean +- SD)
Mean of est. Ln prob of data
K

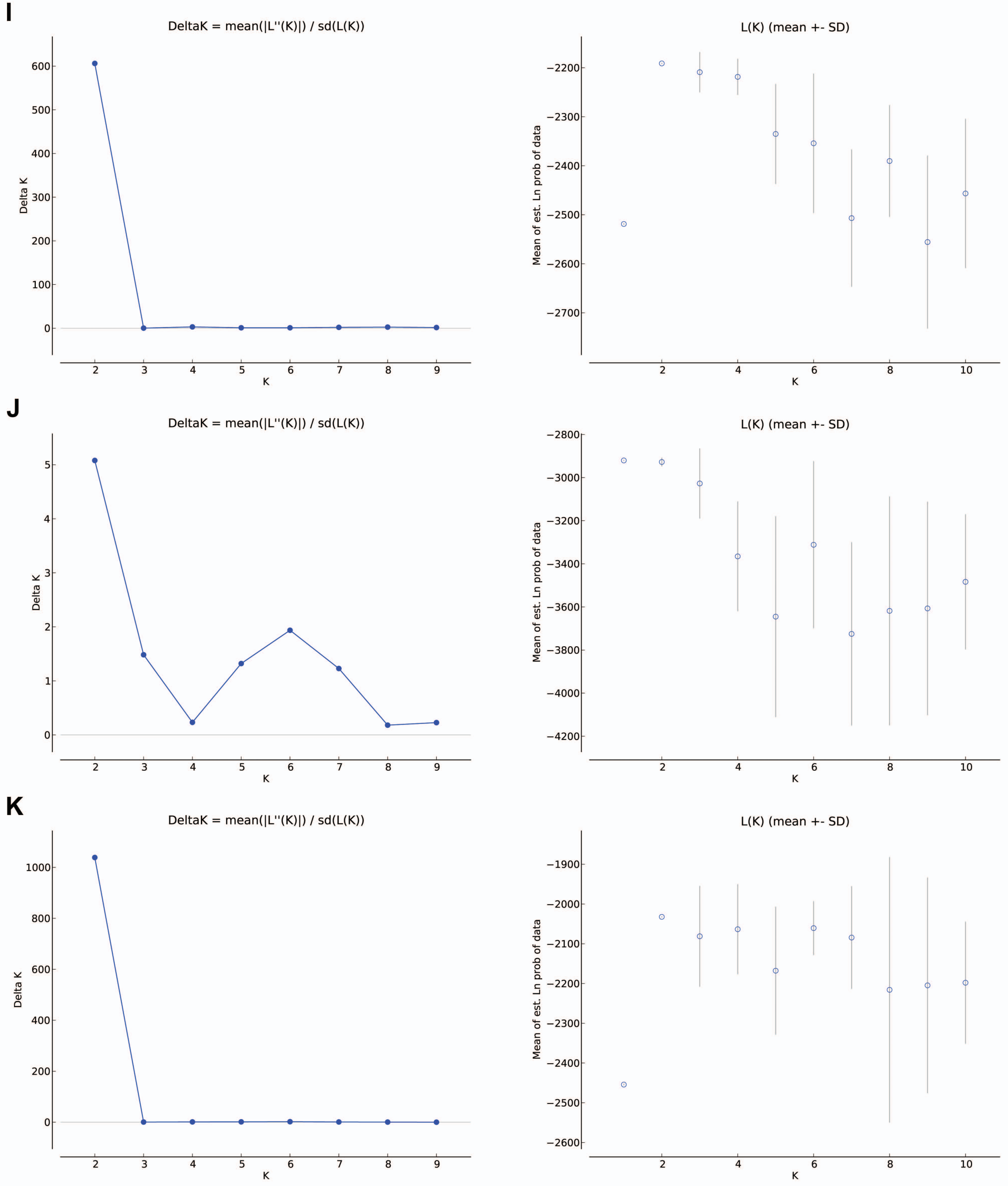
I
DeltaK = mean(|L''(K)|) / sd(L(K))
Delta K
K
L(K) (mean +- SD)
Mean of est. Ln prob of data
K
J
DeltaK = mean(|L''(K)|) / sd(L(K))
Delta K
K
L(K) (mean +- SD)
Mean of est. Ln prob of data
K
K
DeltaK = mean(|L''(K)|) / sd(L(K))
Delta K
K
L(K) (mean +- SD)
Mean of est. Ln prob of data
K

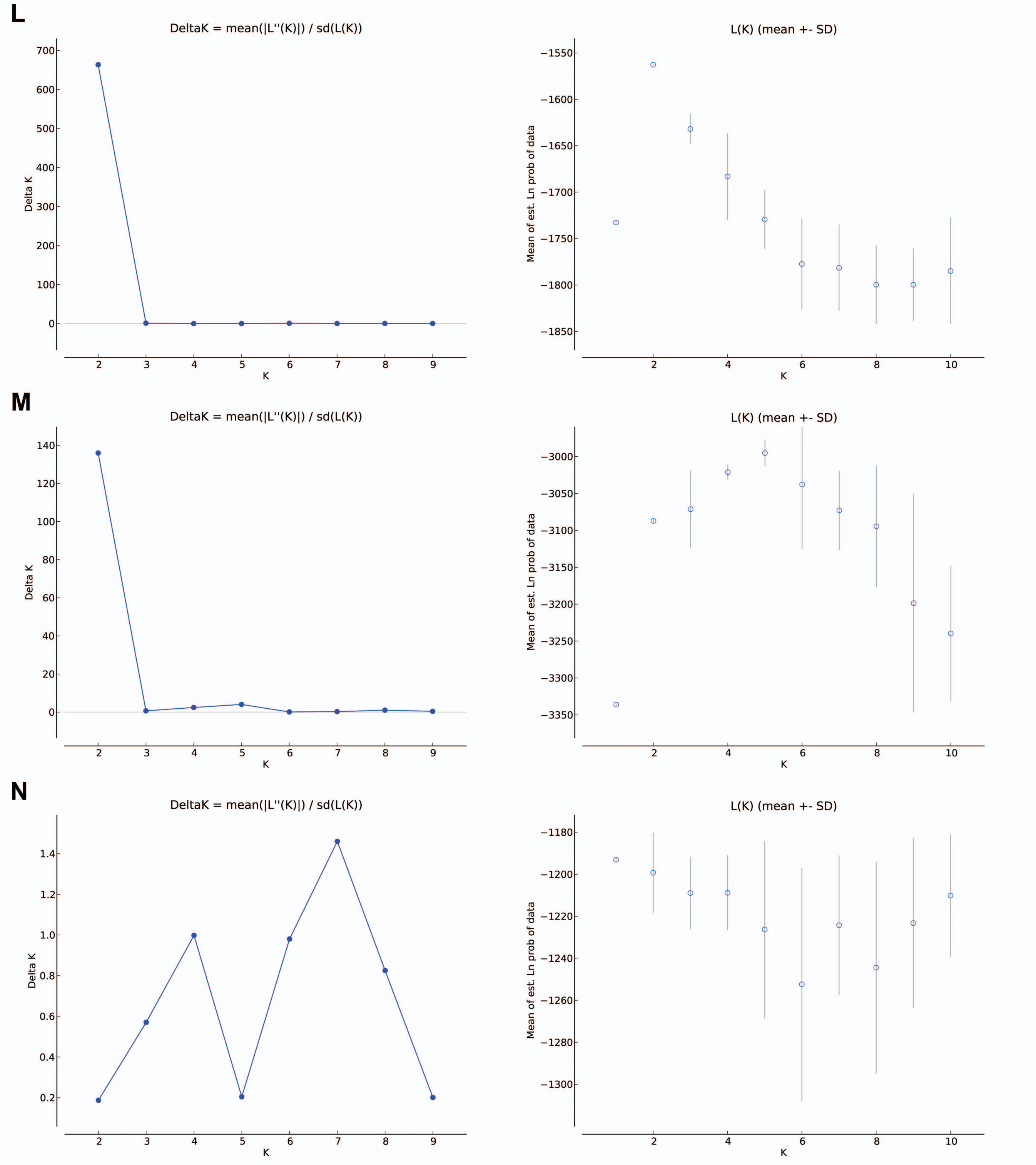
L
DeltaK = mean(|L''(K)|) / sd(L(K))
Delta K
K
L(K) (mean +- SD)
Mean of est. Ln prob of data
K
M
DeltaK = mean(|L''(K)|) / sd(L(K))
Delta K
K
L(K) (mean +- SD)
Mean of est. Ln prob of data
K
N
DeltaK = mean(|L''(K)|) / sd(L(K))
Delta K
K
L(K) (mean +- SD)
Mean of est. Ln prob of data
K

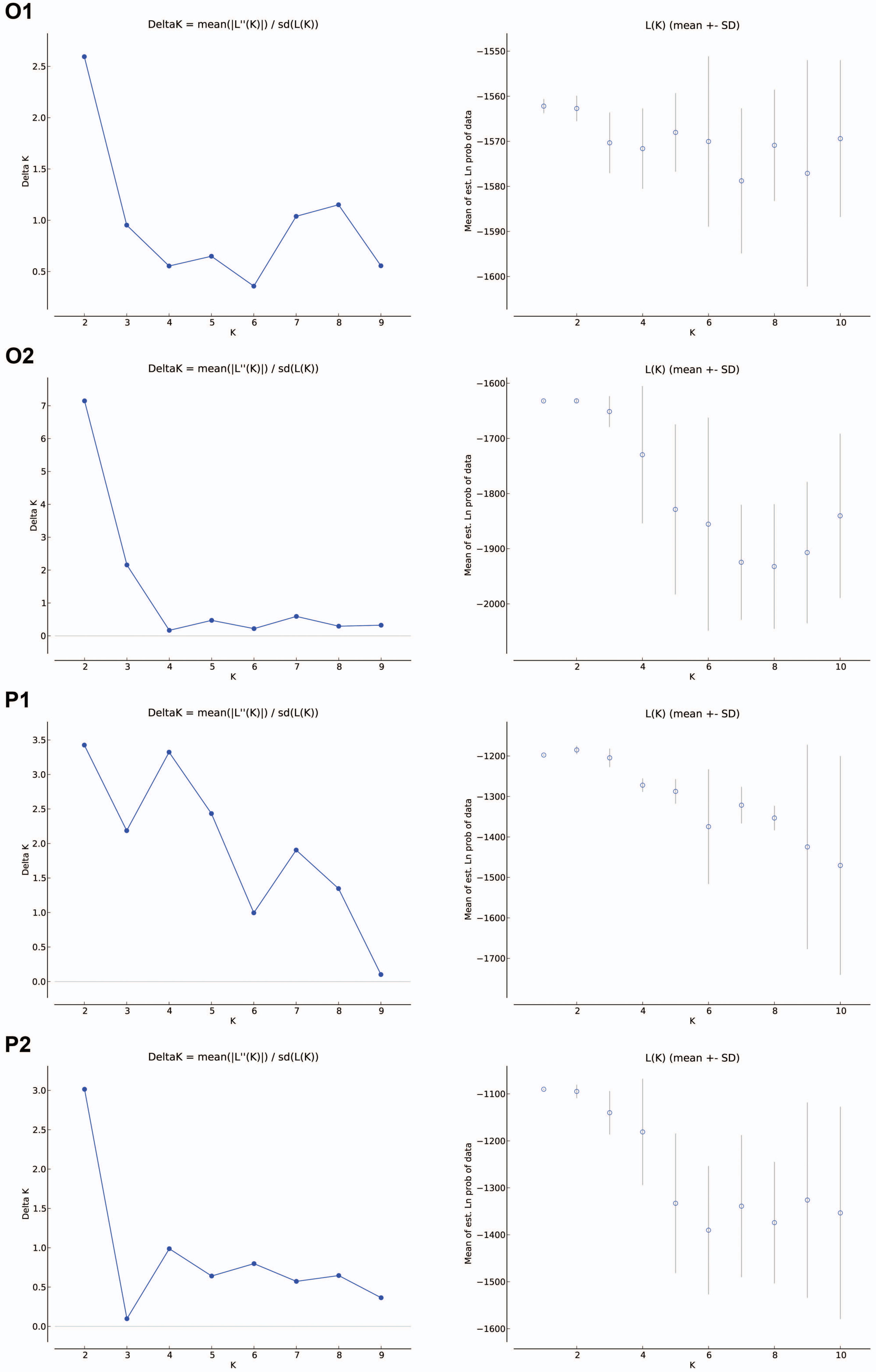
O1
DeltaK = mean(|L''(K)|) / sd(L(K))
L(K) (mean +- SD)
Delta K
Mean of est. Ln prob of data
K
O2
DeltaK = mean(|L''(K)|) / sd(L(K))
L(K) (mean +- SD)
Delta K
Mean of est. Ln prob of data
K
P1
DeltaK = mean(|L''(K)|) / sd(L(K))
L(K) (mean +- SD)
Delta K
Mean of est. Ln prob of data
K
P2
DeltaK = mean(|L''(K)|) / sd(L(K))
L(K) (mean +- SD)
Delta K
Mean of est. Ln prob of data
K

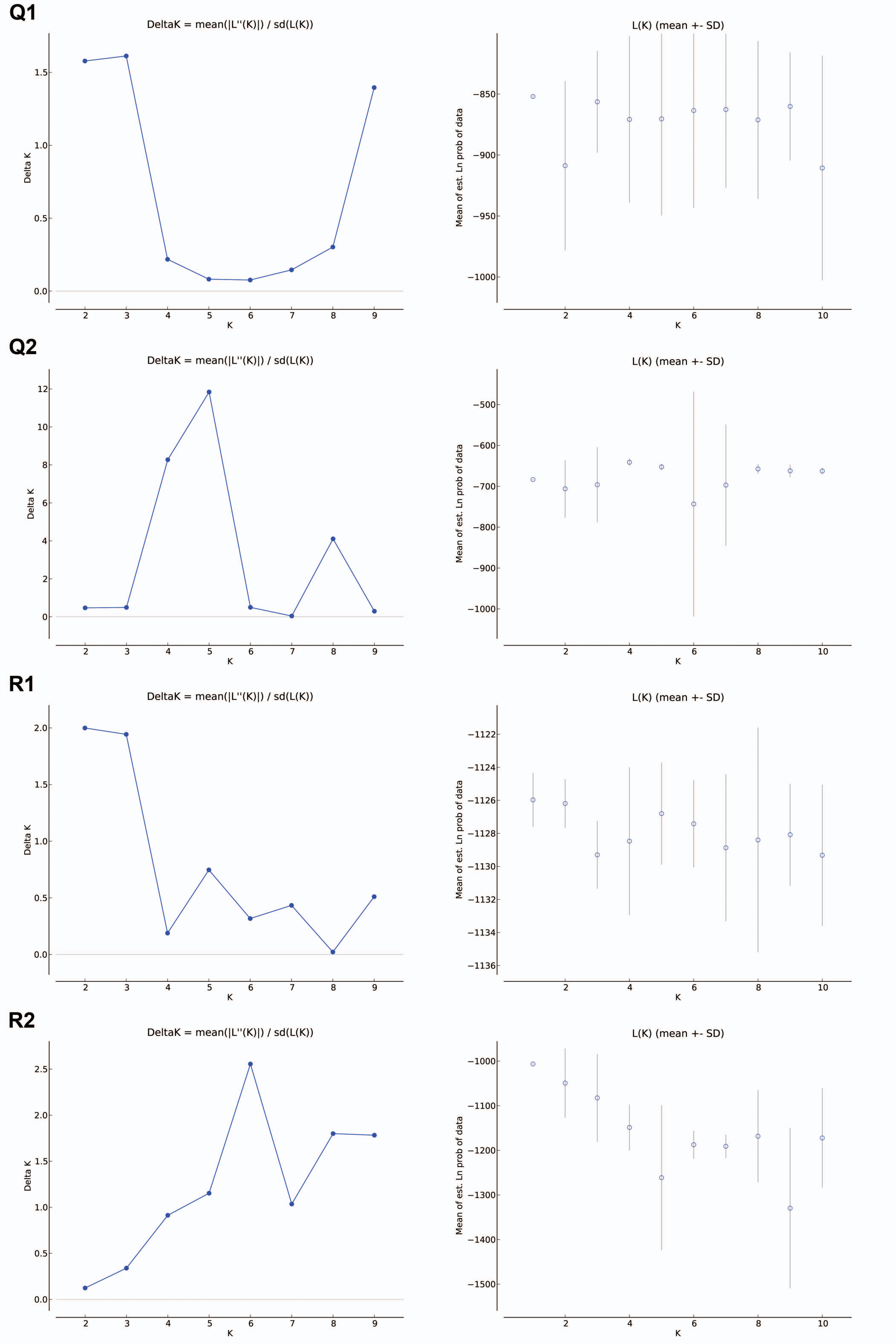
Q1
DeltaK = mean(|L''(K)|) / sd(L(K))
Delta K
K
L(K) (mean +- SD)
Mean of est. Ln prob of data
K
Q2
DeltaK = mean(|L''(K)|) / sd(L(K))
Delta K
K
L(K) (mean +- SD)
Mean of est. Ln prob of data
K
R1
DeltaK = mean(|L''(K)|) / sd(L(K))
Delta K
K
L(K) (mean +- SD)
Mean of est. Ln prob of data
K
R2
DeltaK = mean(|L''(K)|) / sd(L(K))
Delta K
K
L(K) (mean +- SD)
Mean of est. Ln prob of data
K

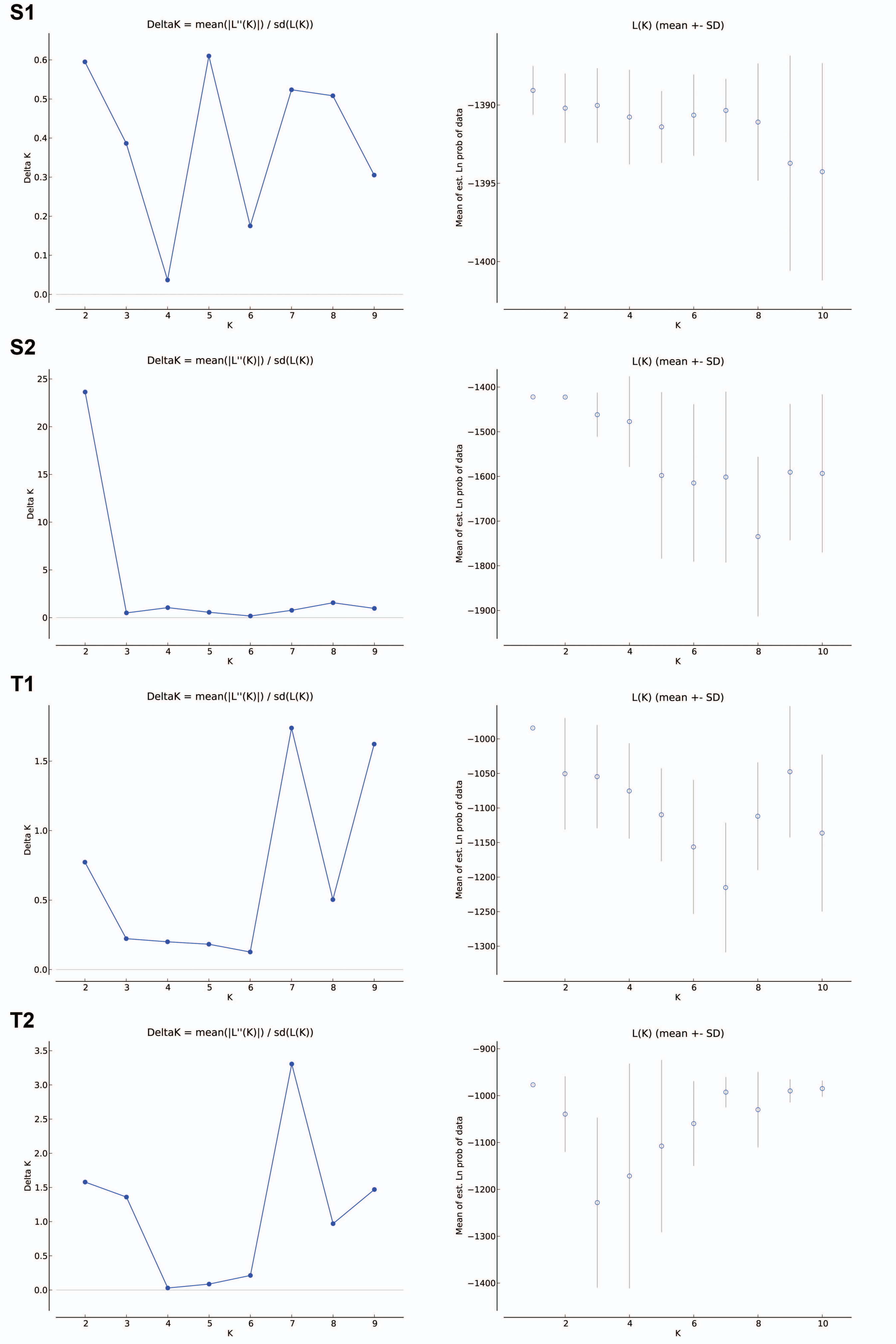

S1
DeltaK = mean(|L''(K)|) / sd(L(K))
Delta K
K
L(K) (mean +- SD)
Mean of est. Ln prob of data
K
S2
DeltaK = mean(|L''(K)|) / sd(L(K))
Delta K
K
L(K) (mean +- SD)
Mean of est. Ln prob of data
K
T1
DeltaK = mean(|L''(K)|) / sd(L(K))
Delta K
K
L(K) (mean +- SD)
Mean of est. Ln prob of data
K
T2
DeltaK = mean(|L''(K)|) / sd(L(K))
Delta K
K
L(K) (mean +- SD)
Mean of est. Ln prob of data
K

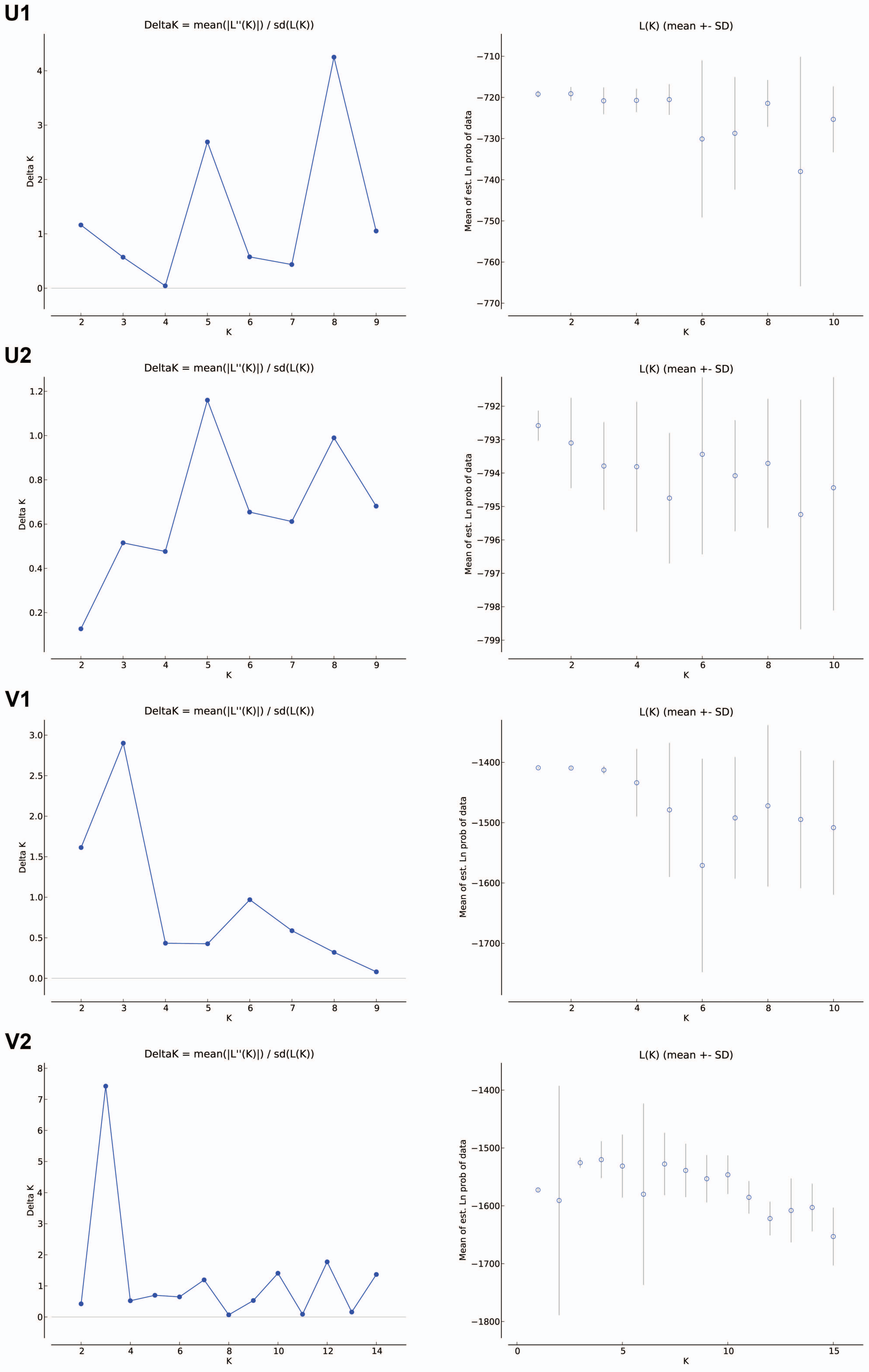
U1
DeltaK = mean(|L''(K)|) / sd(L(K))
Delta K
K
L(K) (mean +- SD)
Mean of est. Ln prob of data
K
U2
DeltaK = mean(|L''(K)|) / sd(L(K))
Delta K
K
L(K) (mean +- SD)
Mean of est. Ln prob of data
K
V1
DeltaK = mean(|L''(K)|) / sd(L(K))
Delta K
K
L(K) (mean +- SD)
Mean of est. Ln prob of data
K
V2
DeltaK = mean(|L''(K)|) / sd(L(K))
Delta K
K
L(K) (mean +- SD)
Mean of est. Ln prob of data
K
