# Supplementary material for: Genetic patterns in Neotropical Magnolias (Magnoliaceae) using de novo developed microsatellite markers
Source: Heredity (Edinb). 2018 Oct 27;122(4):485–500. doi: 10.1038/s41437-018-0151-5 (PMC6460770; doi:10.1038/s41437-018-0151-5)
Supplement: Supplementary file 6 — Supplementary Figure S6 [file 41437_2018_151_MOESM6_ESM.pdf]

Supplementary Figure S6 Confidence intervals of the pairwise F_{ST} values from Table 4. **FSTP** = pairwise F_{ST} (Weir and Cockerham, 1984). White data points represent infraspecific pairwise F_{ST} values. Black data points represent supraspecific pairwise F_{ST} values. **A** dataset 1 which comprises 340 individuals representing 17 populations, genotyped for all 63 microsatellite markers where possible, including the assumed monomorphic data (See Supplementary Table S2: categories A, B and C). **B** dataset 2 which comprises 340 individuals representing 17 populations, genotyped for all 63 microsatellite markers where possible, excluding the assumed monomorphic data (See Supplementary Table S2: categories A and B). **C** dataset 3 which comprises 260 individuals representing 13 populations of the 8 taxa of the section *Talauma* subsection *Splendentes* (See Table 1: Class. = TAS), genotyped for 10 microsatellite markers (See Supplementary Table S2: marker names indicated with an asterisk).

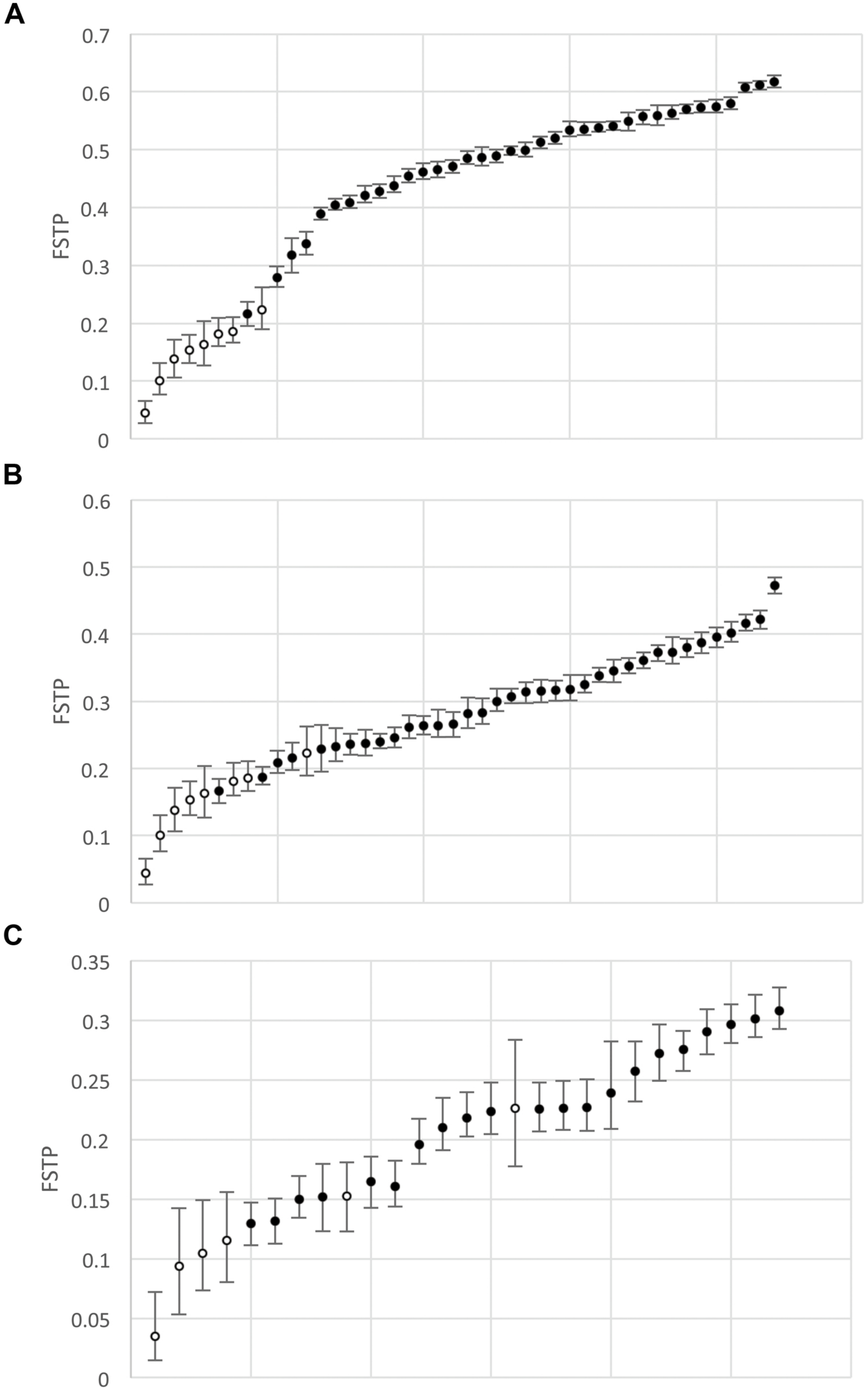

A
FSTP
0.7
0.6
0.5
0.4
0.3
0.2
0.1
0
B
FSTP
0.6
0.5
0.4
0.3
0.2
0.1
0
C
FSTP
0.35
0.3
0.25
0.2
0.15
0.1
0.05
0
