# Supplementary material for: Genetic patterns in Neotropical Magnolias (Magnoliaceae) using de novo developed microsatellite markers
Source: Heredity (Edinb). 2018 Oct 27;122(4):485–500. doi: 10.1038/s41437-018-0151-5 (PMC6460770; doi:10.1038/s41437-018-0151-5)
Supplement: Supplementary file 7 — Supplementary Figure S7 [file 41437_2018_151_MOESM7_ESM.pdf]

Supplementary Figure S7 Mantel tests. **GGD** = Geographic Distance, **FSTP** = pairwise F_{ST} (Weir and Cockerham, 1984). **A** dataset 1 which comprises 340 individuals representing 17 populations, genotyped for all 63 microsatellite markers where possible, including the assumed monomorphic data (See Supplementary Table S2: categories A, B and C). **B** dataset 2 which comprises 340 individuals representing 17 populations, genotyped for all 63 microsatellite markers where possible, excluding the assumed monomorphic data (See Supplementary Table S2: categories A and B). **C** dataset 3 which comprises 260 individuals representing 13 populations of the 8 taxa of the section *Talauma* subsection *Splendentes* (See Table 1: Class. = TAS), genotyped for 10 microsatellite markers (See Supplementary Table S2: marker names indicated with an asterisk).

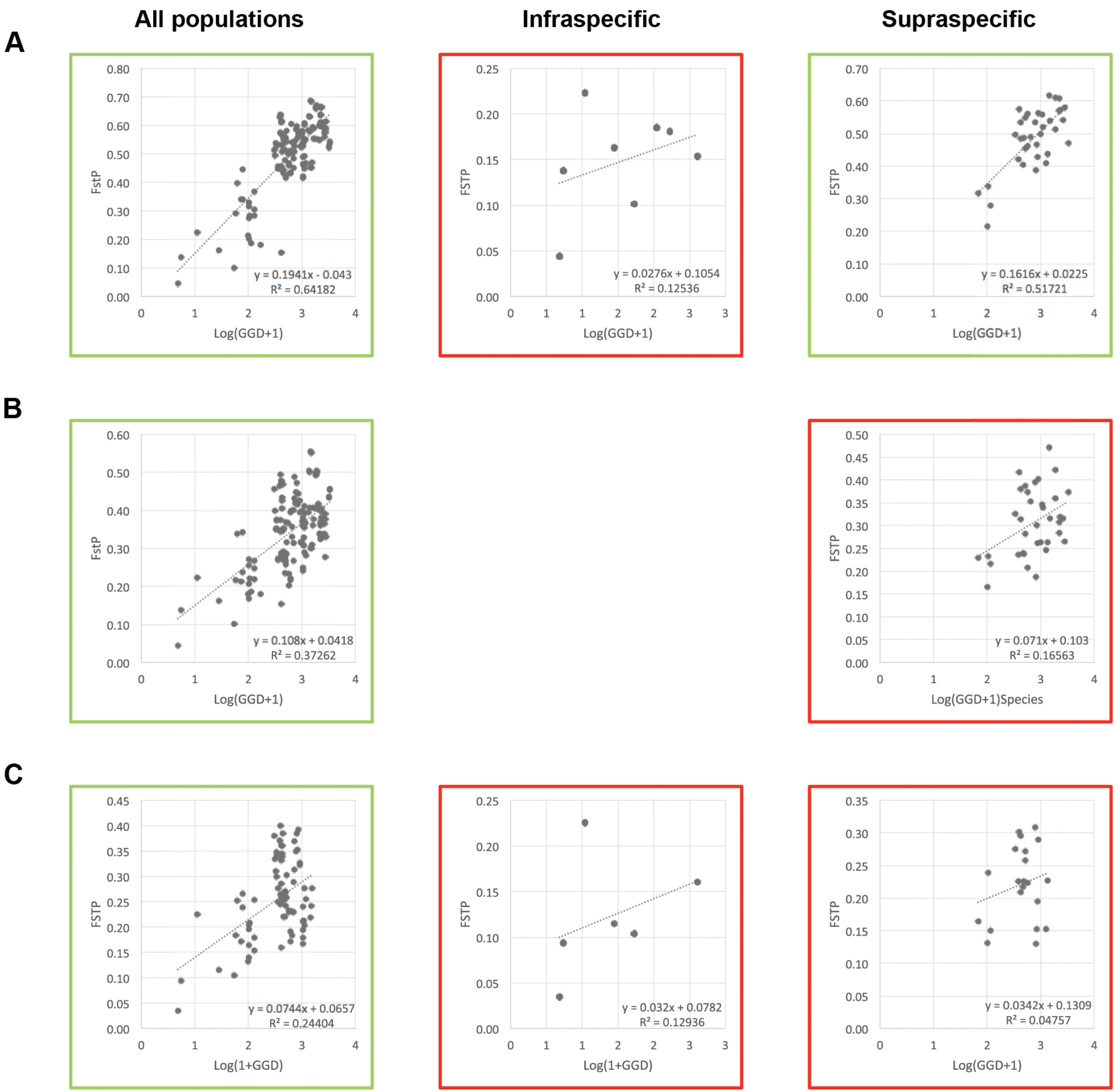

All populations
Infraspecific
Supraspecific
A
B
C
FstP
FSTP
Log(GGD+1)
Log(1+GGD)
Log(GGD+1)Species
y = 0.1941x - 0.043
R² = 0.64182
y = 0.0276x + 0.1054
R² = 0.12536
y = 0.1616x + 0.0225
R² = 0.51721
y = 0.108x + 0.0418
R² = 0.37262
y = 0.071x + 0.103
R² = 0.16563
y = 0.0744x + 0.0657
R² = 0.24404
y = 0.032x + 0.0782
R² = 0.12936
y = 0.0342x + 0.1309
R² = 0.04757
